# 3D morphable systems via deterministic microfolding for vibrational sensing, robotic implants, and reconfigurable telecommunication

**DOI:** 10.1126/sciadv.ade0838

**Published:** 2022-12-21

**Authors:** Lin Zhang, Zongwen Zhang, Hannah Weisbecker, Haifeng Yin, Yihan Liu, Tianhong Han, Ziheng Guo, Matt Berry, Binbin Yang, Xu Guo, Jacob Adams, Zhaoqian Xie, Wubin Bai

**Affiliations:** ^1^ Department of Applied Physical Sciences, University of North Carolina, Chapel Hill, NC 27514, USA.; ^2^ State Key Laboratory of Structural Analysis for Industrial Equipment, Department of Engineering Mechanics, DUT-BSU Joint Institute, Dalian University, Dalian 116024, P.R. China.; ^3^ Ningbo Institute of Dalian University of Technology, Ningbo 315016, P.R. China.; ^4^ Department of Biology, University of North Carolina, Chapel Hill, NC 27514, USA.; ^5^ MCAllister Heart Institute Core, University of North Carolina, Chapel Hill, NC 27514, USA.; ^6^ Joint Department of Biomedical Engineering, North Carolina State University, Raleigh, NC 27606, USA.; ^7^ Department of Chemistry, University of North Carolina, Chapel Hill, NC 27514, USA.; ^8^ Department of Chemical and Biomolecular Engineering, North Carolina State University, Raleigh, NC 27606, USA.; ^9^ Department of Electrical and Computer Engineering, North Carolina Agricultural and Technical State University, Greensboro, NC 27411, USA.; ^10^ Department of Electrical and Computer Engineering, North Carolina State University, Raleigh, NC 27606, USA.

## Abstract

DNA and proteins fold in three dimensions (3D) to enable functions that sustain life. Emulation of such folding schemes for functional materials can unleash enormous potential in advancing a wide range of technologies, especially in robotics, medicine, and telecommunication. Here, we report a microfolding strategy that enables formation of 3D morphable microelectronic systems integrated with various functional materials, including monocrystalline silicon, metallic nanomembranes, and polymers. By predesigning folding hosts and configuring folding pathways, 3D microelectronic systems in freestanding forms can transform across various complex configurations with modulated functionalities. Nearly all transitional states of 3D microelectronic systems achieved via the microfolding assembly can be easily accessed and modulated in situ, offering functional versatility and adaptability. Advanced morphable microelectronic systems including a reconfigurable microantenna for customizable telecommunication, a 3D vibration sensor for hand-tremor monitoring, and a bloomable robot for cardiac mapping demonstrate broad utility of these assembly schemes to realize advanced functionalities.

## Teaser

A microfolding strategy realizes kirigami at nanoscale to form 3D shape-morphable microelectronic systems in freestanding forms.

## INTRODUCTION

Structural engineering that overcomes intrinsic limits of bulk materials pivots a cascading collection of previously unidentified opportunities in biomedical devices (*1*, *2*), robotic systems (*3*, *4*), microelectronics (*5*, *6*), microelectromechanical systems (MEMSs) (*7*, *8*), and metamaterials (*9*, *10*). In particular, morphing mesostructures in three dimensions (3D) not only offers multidimensional control to precisely tune material functions on demand (*6*, *11*, *12*) but also breaks the repulsive barriers for heterogeneous materials to coherently integrate (*13*, *14*), for a leveraged combination of properties beyond those of the individual components (*14*, *15*). Traditional routes to 3D micro- and nanostructures, including ion-beam lithography, layer-by-layer growth, multiphoton lithography, printing-based fabrication, and holographic lithography, which offer high precision in 3D structural formation, are, however, often limited to enable structural morphability for certain high-performance materials such as monocrystalline silicon (Si) nanomembrane (*16*–*19*). Recently developed methods exploit concepts in self-assembly (*20*, *21*) and mechanically guided assembly (*5*, *22*, *23*) to address this limitation with remarkable capability of compatible integration into modern planar technologies and associated thin-film deposition and processing techniques established in semiconductor industry. However, strong reliance on a planar base or template for anchoring precludes the implementation of those approaches in a broader horizon of applications, especially in making reconfigurable medical devices interfaced with biological tissues (e.g., catheter probes and surgical robots) (*24*, *25*). The development of schemes for realizing morphable 3D mesostructures that can enrich classes of materials and designs of devices found in existing forms of electronics, optoelectronics, and MEMSs remains to be a central breakpoint for prevously unknown device capabilities and applications. Furthermore, concepts of origami and kirigami (*26*, *27*), infiltrated in some existing schemes (*7*, *23*, *28*), are yet to unleash substantial potentials, via releasing multidimensional freedoms of folding, in forming diverse morphable mesostructures in 3D.

**Fig. 1.**
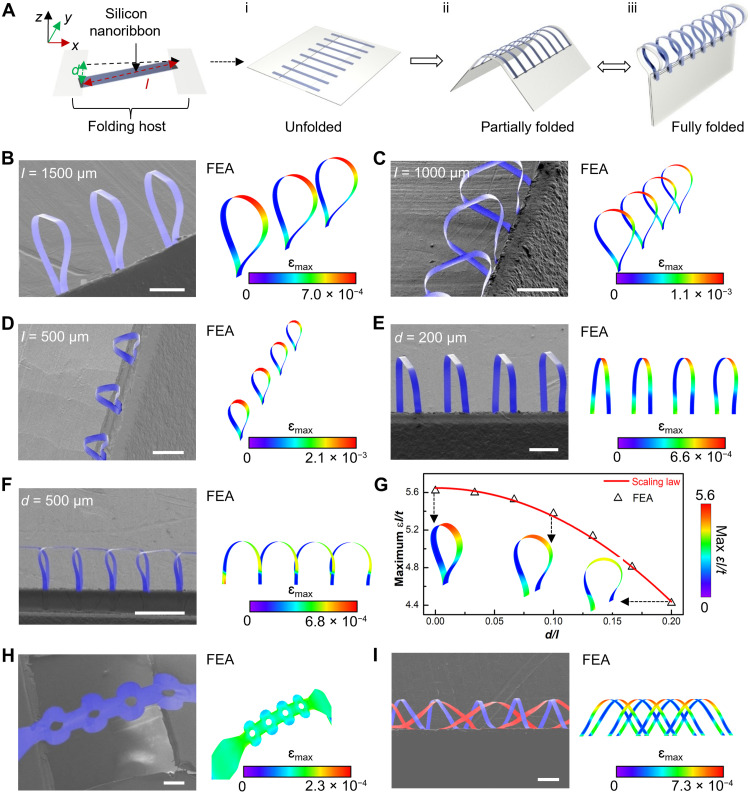
Microfolding assembly of 3D microstructures of monocrystalline Silicon. (**A**) Schematic illustration highlighting the microfolding process, which includes the following: (i) transfer printing to form a hinge structure comprising a 2D precursor and a folding host; the parameters *l* and *d* denote the length of the Si nanoribbon and the distance between the two bonding sites along the *y* axis, respectively, defining the primary dimensional parameters of the integrated system. (ii) Partial folding to form various transitional states of 3D microstructures; the inclination angle (θ; fig. S1C) of the folding host sheet defines the degree of folding. (iii) Full folding (θ = 90°) to form the final state of 3D folded microstructures suspended on the edge of the folding host. (**B** to **F**) Colorized scanning electron microscopy (SEM) images and corresponding finite element analysis (FEA) results of 3D hoops from 2D filamentary serpentine nanoribbons of monocrystalline Si. Aligning 2D Si nanoribbons vertically to the trench edge of a folding host enables precise control of curvatures of the resultant 3D Si hoops by varying the length (*l*) of the Si. (B) *l* = 1500 μm; (C) *l* = 1000 μm; (D) *l* = 500 μm. The dimensional parameter *d* dictates the orientation of the resultant 3D Si hoops. (E) *d* = 200 μm; (F) *d* = 500 μm. (**G**) The scaling law and FEA results of the dimensionless parameter ε*l/t* (defined in note S1) versus *d/l* for *w/l* = 0.05 for 3D Si hoops. Here, *w* and *t* denote width and thickness of the individual nanoribbon, respectively. (**H**) Colorized SEM image and corresponding FEA result of a 3D porous bracelet of monocrystalline Si, with θ ≈ 22.5°. (**I**) Colorized SEM image and corresponding FEA result of a 3D double helix from folding two layers of 2D filamentary serpentine nanoribbons of Si. The top and bottom layers of Si ribbons are colorized blue and red, respectively. Scale bars, 200 μm.

Here, we present a set of strategies and designs that realize deterministic origami at microscale to establish morphable 3D microelectronic systems with a broad range of materials including monocrystalline Si and metallic nanomembranes as well as their hybrid integration (Fig. 1A). The schemes start with integrating a 2D precursor with a folding host through a transfer printing process. Strategically bending the host at various degrees of angle translates the origami effect to the guest 2D precursor into a specially engineered 3D mesostructure. Such host-guest coevolution precisely alters structural reconfigurations through macroscopic folding registrations, angles, and directions of the host to navigate folding trajectories of the microscopic guest precursor toward a broad range of geometrically distinct mesostructures in 3D. Notable outcomes lead to multidimensional control of structural formation and unconventional architectures such as an inverted pyramid of monocrystalline Si sitting on an edge, freestanding microscale cages of gold (Au), and other more than 40 examples of complex 3D forms across various high-performance materials and length scales, which, collectively, present qualitative distinguishment beyond the scope of previously reported strategies in achieving morphable 3D mesostructures. In particular, it provides easy access to a broad range of freestanding 3D mesostructures fully or partially folded. Fundamental studies of the strain distribution, structural stability, and folding behaviors exhibited in the microfolding process reported here establish general rationales for designing 3D morphable mesostructures with distinct, tunable topologies. Moreover, applications in reconfigurable microantennas for wearable telecommunications, 3D vibration sensors for hand-tremor monitoring, and bloomable robots for cardiac mapping highlight the broad utility and scalability of transformable 3D systems realized by the deterministic microfolding.

## RESULTS

### Fabrication scheme and design principle for morphable 3D mesostructures

Figure 1 and fig. S1 present the assembly strategy for constructing 3D mesostructures made of monocrystalline SiNM via deterministic microfolding. The scheme begins with planar micro/nanofabrication of an array of 2D filamentary Si nanoribbons (thickness, 200 nm; width, 50 μm) with an array periodicity of 250 μm (fig. S1A). A multilayer design consisting of the substrate [e.g., polydimethylsiloxane (PDMS)], a sacrificial layer [e.g., poly(methyl methacrylate) (PMMA)], and an adhesive layer [e.g., poly(lactic-*co*-glycolic acid) (PLGA)] with a lithographically defined trench in a precisely controlled geometry serves as a folding host that guides the microfolding assembly process (fig. S1B). Transfer printing of the 2D Si nanoribbons at temperature 70°C with aligned registration onto the folding host leads to the Si nanoribbons being suspended across the trench (Fig. 1Ai). Relatively weak van der Waals forces dictate interfacial interactions between the Si precursor and the PLGA film, thus allowing undisturbed disengagement of the folding host upon completion of the microfolding process to form freestanding 3D mesostructures (details appear in Materials and Methods). Folding the host substrate along the main axis of the trench initiates shape transformation of the 2D precursors, resulting in the 3D folded microstructures (Fig. 1A). Besides the intrinsic design parameters including the length *l*, width *w*, and thickness *t* of the Si nanoribbon, Fig. 1A and fig. S1C define two other primary dimensional parameters that affect the resultant 3D configuration, namely, the distance (labeled as *d*, along the *y* axis) between two bonding sites and the folding angle (the inclination angle of the folding host sheet), respectively. Notably, the microfolding assembly allows additional dimensional freedom of reconfiguration via the folding angle to alter the 3D mesostructures reversibly (Fig. 1A, ii and iii), where the folding host based on a multilayer design with a substrate layer, a sacrificial layer, and an adhesive layer enables freestanding formation with precise preservation of the resultant 3D mesostructures (fig. S1D), both partially and fully folded, depending on the thickness of the substrate layer. Notably, the microfolding strategy offers easy access to a broad range of freestanding 3D mesostructures via strategically initiating bonding at the folding edge and subsequently dissolving the sacrificial layer of the folding host. Figure 1B and fig. S2 show scanning electron microscopy (SEM) images of suspended 3D Si hoops at a fully folded state (θ = 90°) transformed from 2D filamentary Si nanoribbons (ribbon width *w*, 50 μm; thickness *t*, 200 nm; length *l*, 1500 μm; folding parameter *d*, 0 μm). The resultant 3D Si structures suspended from an edge differ qualitatively from in-plane buckling patterns using a prestrained elastic substrate (*23*, *29*, *30*) and are challenging to construct using 3D printing technology (*31*, *32*). On the basis of the finite element analysis (FEA; Fig. 1B), the deformation and corresponding strain distribution of 3D mesostructures formed via the deterministic folding process are predicted (details appear in Materials and Methods).

The lengths, *l*, of the Si nanoribbons (as defined in fig. S3A) dictate curvatures of the resultant 3D Si hoops (Fig. 1, B to D). Increasing *l* from 200 to 1500 μm while fixing *d* to 0 μm effectively increases radius of curvature of the 3D hoops, approximately from 30 to 240 μm, as shown in Fig. 1 (B to D) and fig. S3 (B to D). Moreover, the dimensional parameter *d* (fig. S4A) determines the degree of twisting of the 3D Si hoops. Representative examples include inverted pyramids (Fig. 1E and fig. S4B) and side-by-side archways (Fig. 1F and fig. S4C) with corresponding parameter *d* values of 200 and 500 μm, respectively. The FEA simulation captures the magnitudes and distributions of the maximum principal strain (ɛ_max_) in the Si mesostructures. Figure 1 (B to F) shows slight visual discrepancies between the experiments and FEA simulations, while the discrepancies become negligible when using polyimide (PI) nanoribbons instead of the Si nanoribbon, as shown in fig. S3E. We speculate that this phenomenon is caused possibly by local buckling due to the residual anchors on the edge of the 2D Si precursor, which is resulted from the transfer printing technique, so that thin strips of residual photoresist are tethered on the edges of the Si nanoribbon, leading to a slight downward bending along the short side. Therefore, the experiment shows the folded Si ribbons with a relatively flat top. Notably, this highlights that the microfolding strategy developed in our study opens the possibility to modulate 3D structures at nanoscale. Removing the residual photoresists on the edges of the Si nanoribbons using oxygen-plasma etching effectively prevents them from bending downward, consequently leading to a curved top for the folded 3D hoops (fig. S3B). Figure S3 (B to D) shows the 3D folded Si hoops with a curved top instead of a flat one, as preparation of the Si nanoribbons includes removal of the residual photoresists. Here, the FEA simplifies the Si ribbons with no short-side bending, which results in slight visual discrepancies between the FEA and experiments. These observations suggest that, for fully folded states (θ = 90^o^), the ɛ_max_ levels are largely determined by the dimensional parameters (*l*, *w*, *t*, and *d*) of the 2D precursors relative to the folding host. To guide design optimization for practical applications and ensure that the ɛ_max_ of 3D structure obtained by microfolding registration strategy of ribbons is below the material failure threshold, a scaling law (see details in note S1) is developed to estimate the ɛ_max_ in the resultant 3D mesostructures (fully folded) for different design parameters (*l*, *w*, and *t*) of the Si nanoribbon and the aforementioned dimensional parameter *d* (fig. S5A). Here, the ɛ_max_ in the filamentary Si nanoribbon is proportional to *t/l* (fig. S5, B to D). For *d/l* and *w/l* ranging from 0 to 0.2 and 0 to 0.1, respectively, the influence of *w/l* on ɛ_max_ is negligible, and the ɛ_max_ decreases monotonically with the decrease of *d/l* due to the increasing curvature radius of the ribbon (Fig. 1G and fig. S5E). This scaling law shows a good agreement with experimental observations and provides an inverse-design route to targeted structural formation with the resultant ɛ_max_ below Si fracture thresholds to avoid potential structural failure. In addition, because the scaling law is independent from the mechanical properties of material, it applies to any material that meets the above dimensional ranges.

Figure 1H presents an example with an SEM image and corresponding FEA simulation to highlight the versatility of the deterministic microfolding in constructing 3D Si mesostructures. Here, the designed 2D precursor composed of a line of Si rings undergoes an out-of-plane folding process and then yields a 3D mesostructure resembling a porous bracelet. Furthermore, this microfolding strategy is compatible to build 3D mesostructures with multilayer 2D precursors via layer-by-layer transfer printing. Figure 1I displays an example of a 3D Si double-helix structure. The schematic illustrations in fig. S6A show the corresponding fabrication process. By careful design of dimensional parameters (e.g., *l* and *d*) for each layer, bilayer 2D precursors consisting of intersected Si nanoribbons pop up coherently without over deformation at the junction regions to form a double helix. The transitional states at various folding angles (22.5°, 45°, 67.5°, and 90°, respectively) captured by both SEM images and the FEA simulations (fig. S6, B to E) indicate that the maximum principal strains, ɛ_max_, remain well below the fracture threshold (~2%) for the constituent Si (*14*).

### Representative microfolded 3D mesostructures with deterministic morphability

**Fig. 2.**
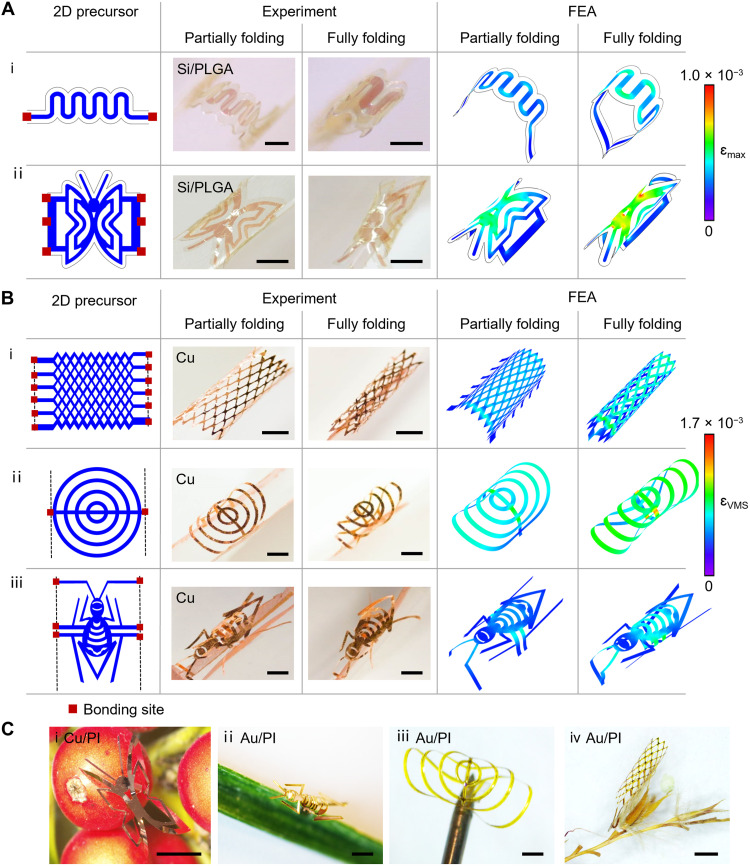
A broad collection of 3D morphable mesostructures based on microfolding assembly. (**A** and **B**) 2D geometries, experimental images, and FEA predictions of partially and fully folded 3D mesostructures. (A) The 3D hair hoop and butterfly structures are made of a bilayer of Si (thickness ~200 nm) and PLGA (thickness ~2 μm). Scale bars, (i) 300 μm and (ii) 1.5 mm. (B) The 3D wire fence, saddle, and spider structures are made of Cu (thickness ~ 1 μm). Scale bars, (i) 500 μm, (ii) 1.0 mm, and (iii) 2.0 mm. (**C**) Optical images of freestanding 3D mesostructures stand on the seed, grass, needle tip, and twig. (i) A butterfly structure is made of a bilayer of Cu nanomembrane (thickness ~150 nm) and PI (thickness ~10 μm); (ii) a spider structure; (iii) a saddle structure; (iv) a wire fence structure. (ii) to (iv) are made of a bilayer of Au nanomembrane (thickness ~ 150 nm) and PI (thickness ~10 μm). Scale bars, (i) 1.5 mm, (ii) 2.5 mm, (iii) 1 mm, and (iv) 500 μm.

**Fig. 3.**
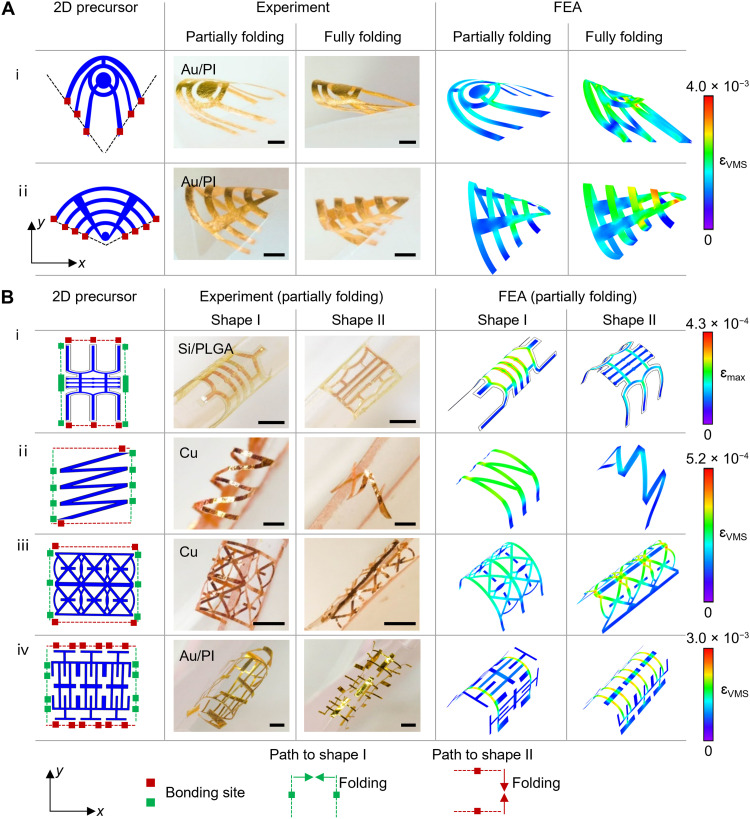
3D morphable mesostructures with deterministic control of geometry via folding registration. (**A** and **B**) 2D precursors, experimental images, and FEA predictions of various 3D mesostructures via configuring folding registration. (A) Angled folding registration to enable various cone-like structures made of a bilayer of Au nanomembrane (thickness ~150 nm) and polyimide (PI; thickness, ~10 μm). Compared with the parallel folding registration shown in Figs. 1 and 2, the angled folding registration highlighted in (A) relies on arranging the pair of trench edges of the host at a predefined angle, 60° and 120°, for i and ii, respectively. Scale bars, 1 mm. (B) Experimental results and FEA predictions highlighting the 3D reconfigurability via switching folding paths for a broad class of 2D precursors. Folding paths with corresponding folding directions aligned along the *y* and *x* axes lead to two distinct 3D configurations, shape I and shape II, respectively, from same 2D precursors. (i) The 3D mesostructures are made of a bilayer of Si (thickness ~200 nm) and PLGA (thickness ~2 μm); (ii and iii) the 3D mesostructures are made of Cu (thickness ~1 μm); (iv) the 3D mesostructures are made of bilayers of Au (thickness ~150 nm) and PI (thickness ~10 μm). Scale bars, (i) 500 μm and (ii to iv) 1 mm.

Through this microfolding scheme, diverse feature sizes and wide-ranging geometries can be achieved in a broad range of materials (Figs. 2 and 3). Figure 2 presents a collection of 3D morphable mesostructures composed of various functional materials (metallic nanomembranes, polymer, and inorganic semiconductor nanomembranes) with geometries ranging from simple to complex states at a submillimeter or millimeter scale. Figure 2A provides representative examples of 3D mesostructures constructed with a Si/biopolymer bilayer (details appear in Materials and Methods). Here, photolithography, etching, transfer printing, and laser cutting define patterns of the bilayer of Si (thickness ~200 nm)/PLGA (thickness ~2 μm), and the microfolding process transforms the 2D precursors into 3D configurations resembling a hair hoop (Fig. 2Ai) and a butterfly (Fig. 2Aii), respectively. Figure 2B presents kirigami-inspired examples using a single layer of Cu (thickness ~1 μm). The first example is a 3D mesh tube that is transformed from a wire fence-patterned 2D precursor (Fig. 2Bi). Figure 2Bii shows a 3D saddle formed from a 2D precursor of concentric circles. The 3D mesostructure in Fig. 2Biii resembles a spider, constructed from a patterned 2D precursor that uses controlled folding to realize the 3D transformations. Figure S7A(i to iv) show additional 3D folded mesostructures that resemble a fishing net, a sliding door, a hollow-out lampshade, and an array of hair hoops, respectively. Notably, 3D mesostructures with folding impact applied selectively at local regions can be achieved via designing 2D precursors with decoupled feature elements and arranging the bonding sites only at a partial set of the feature elements. Figure S7Av shows a butterfly pattern consisting of inner and outer wings with little interconnections between each another. Upon microfolding, the outer wings undergo notable shape transformation, while the inner wings have little shape change due to the asymmetrical concentration of mechanical constraints mainly on the outer wings through the bonding sites. Thereby, the microfolding strategy provides the capability to control the shape evolution locally at targeted regions, expanding the versatility of 3D structural formation. Here, the optical images and FEA simulations (fig. S7A) reveal both the intermediate (partially folded) states and final (fully folded) configurations, indicating high reversibility in the structural transformation. In all cases, the freestanding 3D mesostrutures can be naturally blended with various environments such as the plant seed, grass leaf, needle tip, twig, and even water (Fig. 2C and movie S1), which paves the way for their broad applications.

The aforementioned designs rely on a parallel folding registration, where the trench edges of the folding host are parallel to each other, to enable 3D transformation. Here, configuring folding registration can further expand the design versatility. Figure 3A illustrates examples of 3D cone-like structures relying on angled folding registration. As illustrated in Fig. 3Ai, a lithographically defined bilayer of Au nanomembrane (thickness~150 nm) and PI (thickness ~10 μm) bonded onto a folding host, whose edges are arranged with a predefined angle 60°, forms a jellyfish-like 3D structure. The optical images and FEA simulations capture the transitional and final states of the assembly process that resemble swimming movements of the jellyfish. Moreover, the compatible range of the angle between the trench edges of the folding host could span from acute to obtuse angles. An example based on obtuse-angled folding registration appears in Fig. 3Aii, highlighting the transformation from a 2D bilayer of Au nanomembrane (thickness ~150 nm)/PI (thickness ~10 μm) to an ice cream cone structure via arranging the pair of the trench edges at a predefined angle 120°. Adopting the same folding registration, a flying eagle and a moving insect are demonstrated in fig. S7B (i and ii), respectively.

Furthermore, this microfolding scheme enables 3D reconfigurability by switching folding registrations between various folding axes to generate markedly distinct 3D mesostructures from the same 2D precursors. Figure 3B and fig. S8 present a class of 2D precursors of various materials and/or patterns that can be reshaped between two distinct 3D configurations. Specifically, Fig. 3Bi shows that a Si/PLGA bilayer with a ribbon-shaped geometry folds along the *x* and *y* axes, leading to a turtle shell (shape I) and a shield (shape II), respectively. This design strategy also enables a different set of ribbon-shaped mesostructures constructed with either metallic membranes or bilayers of metal and polymer. Figure 3Bii shows that a zigzag Cu ribbon can form a singular shoelace with the *y*-axis folding, whereas the *x*-axis folding transformation yields a decorated ring. Even in cases of 2D Cu precursors with bilateral symmetry, as illustrated in fig. S8Bi, the resulting 3D mesostructures resemble either diamonds in a series or a fence depending on the folding paths. In addition, the switching folding registration is also applicable to complex morphable 3D mesostructures with hybrid ribbon/circle geometries, as shown in Fig. 3Biii and fig. S8Bii. Figure 3Biv provides 3D mesostructures with geometric complexity constructed in with an Au/PI bilayer in a periodic pattern of ribbons, where a birdcage and spiked sticks are assembled through folding along the *x* and *y* axes, respectively. Moreover, adopting folding registration along dual axes of folding can further program the system into distinct 3D mesostructures. Figure S8C presents an Au/PI bilayer design that achieves a bamboo dragonfly (shape I) and a four-legged footstool (shape II), via single-axis folding and dual-axes folding, respectively. The strains of microfolded configurations for all these cases, shown by FEA simulation, are well below the fracture thresholds of corresponding materials and excellent agreements with the corresponding experimental observations. Compared to reconfiguration methods of 3D mesostructures activated by smart materials including shape memory polymers (*24*), shape memory alloys (*33*), and liquid crystal elastomers (*12*, *34*), under various external stimuli (e.g., thermal, chemical, optical, magnetic, electric, and mechanical strategies) (*35*), the microfolding strategy offers high structural precision, stability, and continuous morphability via a well-controlled folding host.

### 3D morphable dipole microantennas

**Fig. 4.**
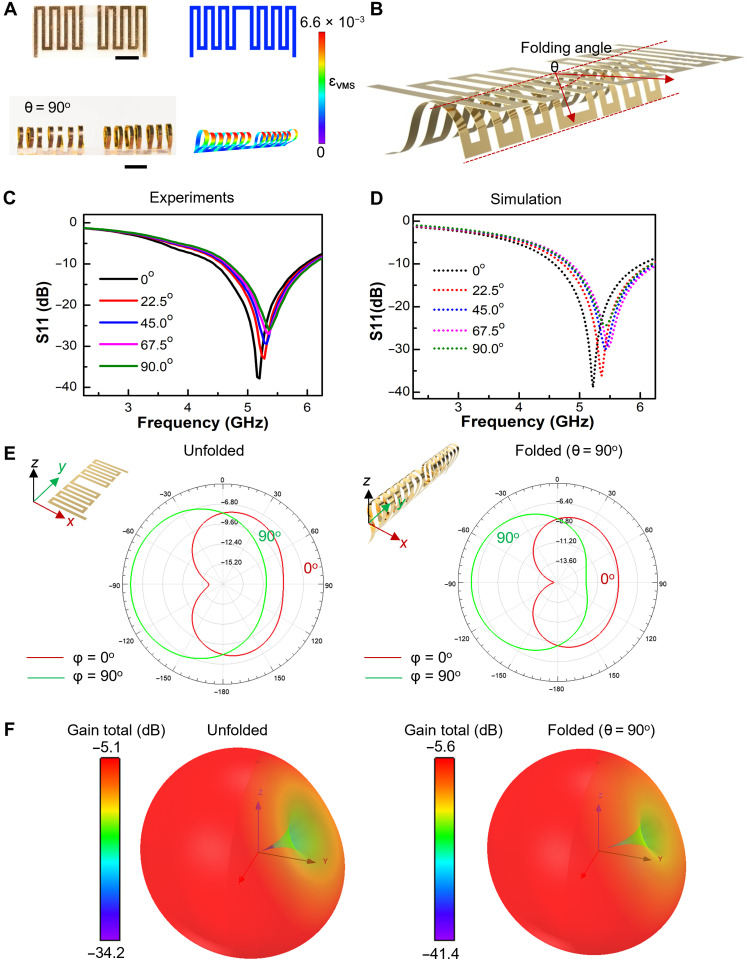
A morphable, dipole microantenna controlled via microfolding. (**A**) Optical images of the 2D and fully folded 3D antennas based on microfolding, and the corresponding FEA results with a color scheme that denotes the magnitude of the equivalent strain based on Von Mises Stress (ɛ_VMS_) in the Cu layer. (**B**) Schematic illustration defining the microfolding angle θ for the 3D folded antenna. The folding angle θ of the 3D antenna (A, bottom) is 90°. (**C** and **D**) Results of experimental measurements (C) and electromagnetic simulation (D) for the return loss (S11) of the 3D microantenna under various folding angles. (**E** and **F**) The simulated 2D (E) and 3D (F) radiation patterns of 2D and fully folded 3D microantennas at 5.2 GHz. Scale bars, 2 mm.

The miniaturization of antennas plays an important role in wireless communication technology (*36*, *37*). In general, the antenna is miniaturized by in-plane configuration design in previous studies (*38*, *39*). The developed origami scheme offers high compatibility with modern planar device technologies and broad versatility in structural design and transformation, which provides an effective way for antenna miniaturization in 3D. Here, we fabricate a reconfigurable dipolar microantenna from a bilayer of copper (Cu) nanomembrane (thickness ~110 nm) with a serpentine pattern and PI base (thickness ~10 μm) (fig. S9A) via the microfolding assembly (details appear in Materials and Methods). Figure 4A shows the 2D and fully folded 3D antennas and the corresponding FEA results with a color scheme that denotes the strain distribution in the Cu layer. Moreover, tuning the folding angle θ defined in Fig. 4B can yield 3D antennas with various folding states (fig. S9B) for performance and structural modulation. The FEA results show the equivalent strain based on Von Mises Stress (ɛ_VMS_) in the Cu layer for all these states is notably smaller than the elastic limit (~0.3%) of Cu (*40*), indicating that the microfolding assembly construction allows stable 3D configurations for the full range of folding angles. Figure 4C shows the reflection coefficient S11 of the antennas with a folding angle ranging from 0° to 90°. It can be observed that the working frequency of 2D microantenna is 5.20 GHz with a minimum S11 of −38 dB. The impedance characteristics based on the measurement and simulation are shown in figs. S10 and S11. When it is fully folded (θ = 90°), the center frequency only slightly shifts to 5.32 GHz with a minimum S11 of −26 dB, which agrees well with the simulation results (Fig. 4D). The resonant frequency keeps mostly unchanged, indicating that 3D microantennas own high robustness, in which the designed operational frequency is insensitive to large folding deformation. The slight impedance change (fig. S10) of the antenna’s structural transformation from 2D to 3D induces negligible change of S11 parameter (Fig. 4, C and D). The simulated 2D and 3D radiation patterns at the frequency of 5.20 GHz for all the folded states are plotted in Fig. 4 (E and F) and fig. S12. All the radiation patterns are omnidirectional and in an irregular dumbbell shape at H- and E-plane, respectively. This suggests that the performance of the microantenna during the 3D transformation has a negligible change in the spatial distribution when the folding angle spans from 0° to 90°. The stable performance of S11 parameter, gain, and radiation pattern of the 3D antennas under various folding configurations indicate that the microfolding registration enables miniaturization of meander-line antennas in 3D and offers the opportunity to adjust their geometry on demand with consistent performance, which is unique compared with existing planar miniturization approaches (*41*) and is useful in different targeted applications ranging from telecommunication to biomedical applications based on implantable miniaturized devices (*42*).

### 3D vibration sensor for monitoring hand tremor

**Fig. 5.**
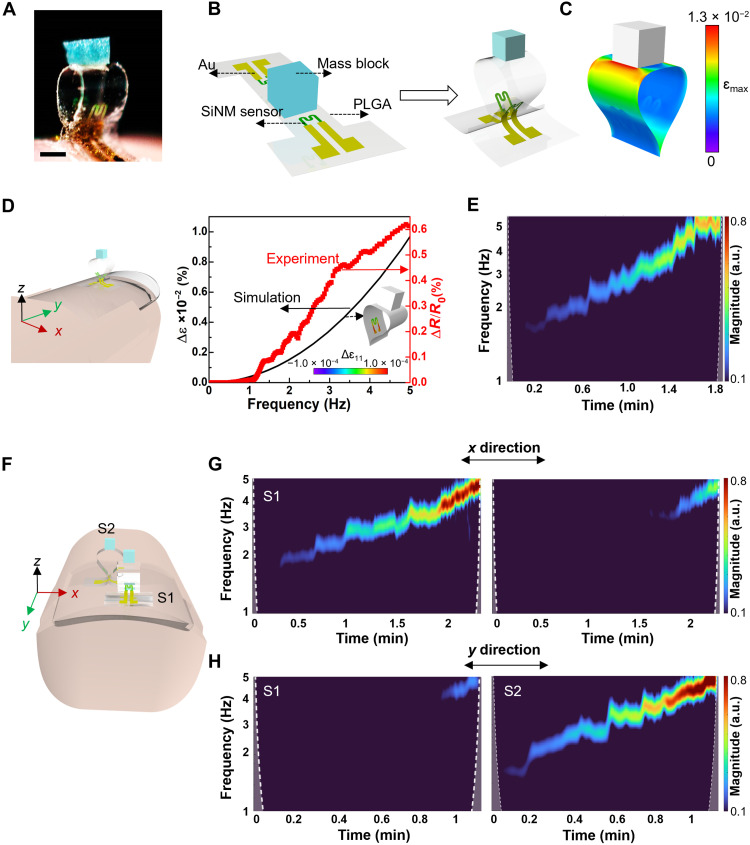
A vibration sensor based on a 3D microfolded mesostructure for monitoring hand tremor. (**A**) Image of a wearable 3D vibration sensor placed on a fingernail. (**B**) Schematic illustration highlighting the microfolding strategy that enables the capability of sensing acceleration. (**C**) The FEA results of the 3D fully folded mesostructure used by the wearable vibration sensor. (**D**) The relative strain change from FEA and the corresponding resistive change of the Si sensor from experimental measurement as a function of vibrational frequency along one direction (*x* axis, as labeled in the schematic illustration on the left). The inset shows the simulated distribution of strain at a vibrational frequency of 3.3 Hz. Here, in the experimental measurement, the linear actuator was used to produce periodic vibrations with programmable frequencies between 0 and 5 Hz. (**E** to **H**) Representative measurements showing signal responses of a 3D vibration sensor placed on a fingernail during various vibrational motions. (E) Time-frequency analysis of signals based on continuous wavelet transform (*60*) showing vibrational intensity at various vibration frequencies ranging from 0 to 5 Hz, covering medical relevance of hand tremor. a.u., arbitrary units. (F to H) Integrating two 3D vibration sensors that are oriented vertically to each other enables directionally resolved measurements of vibrational activities. (F) Schematic illustration showing the multisensor integration for directionally resolved measurements of vibrational activities. (G and H) Frequency spectra showing vibrational activity collected from an integrated pair of vibration sensors [labeled as S1 and S2, respectively, as shown in (F)], which composes a vector data point that defines vibrational directionality. Here, the experiments use finger vibrations with directions along the *x* and *y* axes [as defined in (F)], respectively, for demonstration. Scale bar, 1 mm.

Hand tremors are common symptoms among patients with neurological disorders, especially Parkinson’s disease, and many other disorders involved with anxiety or the overproduction of a certain hormone (*43*). The treatment and monitoring of tremor still represent a substantial challenge for clinicians as tremor is highly variable in its characteristics within a day and over several days (*44*). Here, we fabricate a wearable vibration sensor based on a 3D microfolded mesostructure (fig. S13, A and B), which can accurately capture and assess tremors in real time to evaluate the symptom severity. Figure 5A and fig. S13C show that a small (<4 mm) and lightweight (~9.5 mg) 3D vibration sensor is placed on a fingernail to detect and monitor hand tremors. As illustrated in Fig. 5B, the microfolding strategy enables the transformation from the planar device into a 3D architecture. The construction mainly involves a proof mass (Cu/PDMS composite, 1 mm by 1 mm by 1 mm) and a PLGA ribbon (10 μm in thickness, 2 mm in width, and 7 mm in length) that serves as a supporting substrate for the sensors of Si in a serpentine geometry (200 nm in thickness) and interconnects with associated metal. The FEA results in Fig. 5C show the strain distributions in the mesostructure during the transformation from the planar into the 3D hoop structure used by the wearable vibration sensor. As illustrated in Fig. 5D, the 3D vibration sensor is mounted on the index finger that allows detection of the presence of tremor as the hand moves along different directions. FEA demonstrates the strain change of the Si sensor in the frequency range from 0 to 5 Hz when the displacement excitation with the amplitude of 15 mm along each coordinate axis is applied to the bottom of the 3D vibration sensor. Figure S14 (A to C) indicates that the strain change caused by vibration in the *x* direction is around 1200 times and 20 times of those in the *y* and *z* directions, respectively. Because of the piezoresistive effect, the resistance of the Si sensor is linearly proportional to its strain change, i.e., the vibration in the *x* direction plays a dominant role in the resistive change of the SiNM sensor, which provides theoretical guidance for the design and placement direction of the 3D vibration sensor. In fig. S14D, the appropriate design of the geometry and weight of the mass proof can ensure that the critical frequency (~36 Hz) of Si fracture is distant from the target range of the working frequency (1 to 5 Hz) including the hand rest tremors (3 to 5 Hz) (*45*, *46*). Figure 5D shows the output in terms of a fraction change in resistance (Δ*R*/*R*) as a function of frequency for the *x*-direction vibration. Here, the programmable frequencies between 0 and 5 Hz were controlled by a linear actuator system. As shown in Fig. 5D, both the simulated strain change and measured resistance change of the SiNM sensor show a proportional correlation with the vibrational frequency. Next, the 3D vibration sensor is positioned on the index finger to validate its capability in capturing subtle finger movements. Figure S15 (A to C) summarizes representative time-domain resistive change in response to vibration in different directions at frequencies ranging from 1 to 5 Hz. The corresponding frequency spectra of the sensing signals in Fig. 5E and fig. S15 (D and E) show a higher sensitivity to vibrational frequency in the *x* direction than those in the *y* and *z* directions, denoting the dominant sensitivity of the as-fabricated 3D vibration sensor in a primary direction of vibration, which is consistent with the result as shown in Fig. 5D and fig. S14 (A and B) as labeled in the schematic illustration in Fig. 5D. Figure S15 (F and G) shows little distortion and degradation of signal quality collected from a 3D vibration sensor in response to a 5-Hz vibration along the *x* direction after being operated for 50,000 cycles (each cycle consists of 10 min of cycling vibrations; frequency of 5 Hz and *x* direction), demonstrating the excellent performance reliability of the 3D Si vibration sensor. In addition, the Si sensor with a fully folded configuration undergoing bending-dominated deformation is more sensitive compared to the 3D partially folded one that experiences stretching-dominated deformation. Figure S16 indicates that the 3D vibration sensor based on a fully folded mesostructure offers higher sensitivity than that based on a partially folded mesostructure, thus highlighting the advantage of the microfolding assembly. The broad tunability of sensitivity and sensing range via configuring folding conditions can match performance requirements (vibrational frequency and amplitude) for various practical applications. Furthermore, the orientational selectivity of vibration sensing in the 3D vibration sensor enables differentiation of vibrational directions by integrating multiple 3D vibration sensors. Figure 5F demonstrates that an integrated pair of vibration sensors (S1 and S2) that are oriented vertically to each other is positioned on a finger. As shown in fig. S17 and Fig. 5 (G and H), the vibration sensors S1 and S2 can effectively capture the vibrational frequency and the intensity simultaneously along the *x* and *y* directions, respectively, for trembling movements of the finger. This also indicates that the 3D vibration sensor shows dominant sensitivity in a primary direction, with negligible sensitivity of the redundant vibration from other directions. As a result, the integration of the multiple 3D vibration sensors based on a microfolded 3D mesostructure suggests a potential route to precisely map the hand vibration variety caused by tremor and to assist in the diagnosis and rehabilitation management of the movement disorders.

### Bloomable robot for cardiac depolyment

**Fig. 6.**
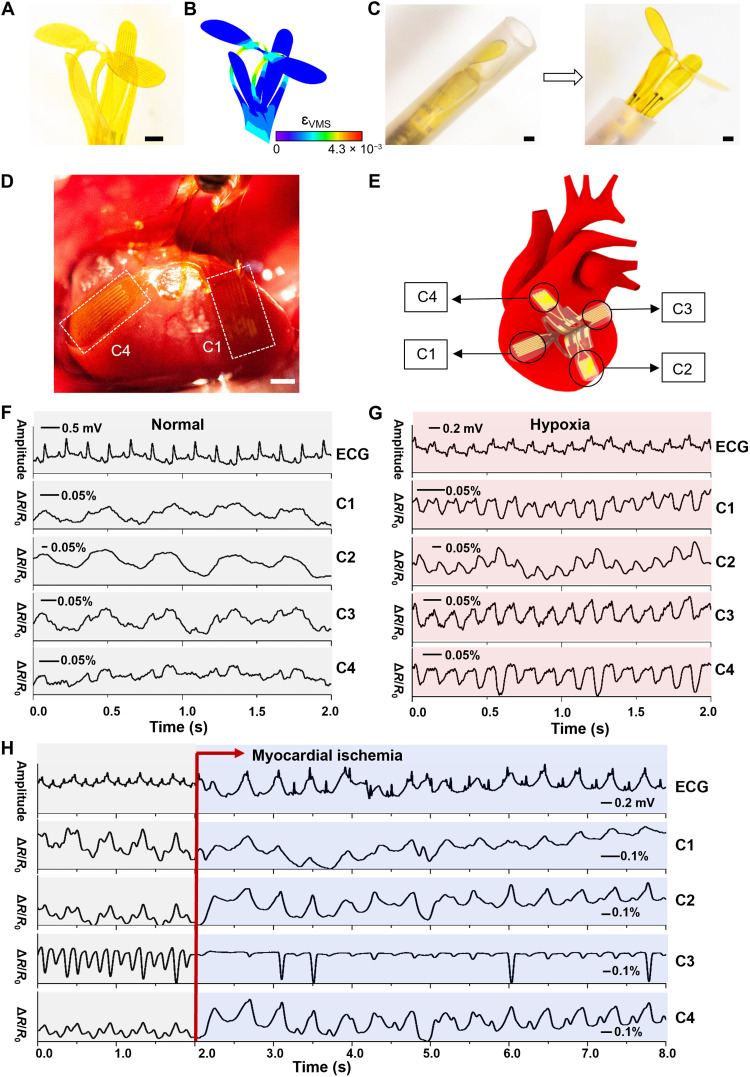
Bloomable robots for cardiac depolyment. (**A**) An optical image and (**B**) the corresponding FEA result of a fully bloomed 3D epicardial bioelectronic robot. (**C**) The optical images showing the blooming process of an epicardial bioelectronic robot from a catheter-integrated structure with minimally invasive modality of intrapericardial insertion, into a 3D flower-like architecture to closely engage geometrically irregular tissue of heart. (**D**) Image and (**E**) schematic illustration of a 3D bioelectronic robot deployed on the epicardial surface of a living mouse heart, where the sensors (labeled as C1, C2, C3, and C4), located in different heart chambers, gather spatially resolved information that enables holistic monitoring of cardiac contractility. The representative measurements of an epicardial bioelectronic robot placed onto a living mouse heart under (**F**) normal conditions (partial pressure of oxygen was 150 mmHg), (**G**) hypoxia condition (partial pressure of oxygen was 100 mmHg), and (**H**) conditions before and after an ST-elevation myocardial ischemia, with real-time comparison of the corresponding ECG. Scale bars, ~1 mm.

Probing epicardial surfaces via minimally invasive approaches can enable real-time, continuous monitoring of contractility of local cardiac tissues, cardiac output, and stroke volume, all of which are essential in discovering and treating heart diseases with enhanced precision and timeliness (*47*, *48*). However, existing monitoring equipment including Doppler echocardiography, magnetic resonance imaging (MRI), and angiography often lacks convenient accessibility because of the need of medical referral, limited availability, high operational cost, and requirement for well-trained technology specialists. Here, we develop a bloomable robot that can be enclosed in a catheter structure (diameter of ~3 mm) with a minimally invasive modality of intrapericardial insertion. Figure S18 presents the planar form of an epicardial bioelectronic system including a precut substrate layer of PI (10 μm in thickness) in a flower-shaped geometry with four petals (fig. S18A), four resistive strain sensors consisting of Au serpentine resistors (50 nm in thickness) lying on the petals separately (fig. S18B), and an encapsulation top layer of parylene (2 μm in thickness) (details appear in Materials and Methods). Figure 6 (A and B) shows the optical image and corresponding FEA result of a fully bloomed 3D epicardial bioelectronic robot obtained from the 2D integrated electronics via the microfolding assembly. As aforementioned, the fully bloomed epicardial bioelectronic robot can be encapsulated into a catheter because of its mechanical softness and deformability; Fig. 6C depicts the blooming process of the epicardial bioelectronic robot from a catheter structure. Here, the flower-like architecture can undergo shape transformation from closure to opening spatially and reversibly as shown in fig. S19. For example, the 3D epicardial bioelectronic robot in a closing state can safely travel in thoracic cavity or through the vein to the heart. Once it reaches the desired location, the catheter is retracted, and the flower structure emerges immediately to engage closely with targeted tissues. Figure 6D and fig. S20B demonstrate a 3D bioelectronic robot with an enhanced interface to geometrically irregular cardiac tissue conformally deployed on the epicardial surface of a living mouse heart. As illustrated in Fig. 6E, multisensors (labeled as C1, C2, C3, and C4) are well aligned on the petals of the device and simultaneously distributed across the four chambers of the heart, gathering spatially resolved information that enables real-time, holistic quantification of cardiac contractility, which can assist diagnosis and treatment of heart conditions (*49*, *50*).

Here, the representative measurements involve signals acquired from a living mouse heart for a variety of conditions including normal condition, hypoxia condition, and ST-elevation myocardial ischemia attack (details appear in Materials and Methods). The sensors stretch and conform in accordance with the relaxation and contraction of myocardium, which resembles the cardiac electrophysiological signals. Moreover, the electrocardiogram (ECG) recordings verify the electrical capture. Figure 6F and fig. S20C present that the as-prepared device enables simultaneous measurement of the specific contraction and relaxation of the right and left atria (RA and LA, respectively), and the right and left ventricles (RV and LV, respectively) under normal beating conditions, where the output features of the sensors will depend on their experienced strain that strongly correlates to their positions on the epicardial surface. As shown in fig. S20C, in each healthy cardiac cycle, pressure in C3 (LA) and C4 (RA) increases as the atria contract, increases slightly as the mitral and tricuspid valves close, and continues to increase through the end of the T wave for C3 due to venous return from the lungs, but C4 pressure remains at baseline throughout atrial diastole. In contrast, pressure in C1 (RV) and C2 (LV) slightly increases as blood flows into the ventricles and rapidly increases during the QRS (Q wave, R wave, and S wave) complex as the ventricles depolarize and contract before decreasing back to baseline as the ventricles repolarize and enter relaxation. In addition, the relative activation delay displayed in C1 (RV) and C4 (RA) compared with that in C2 (LV) and C3 (LA) occurs because the right side of the heart is stimulated before the left, well corresponding to the myocardial locations at which sensors are placed. Moreover, a general hypoxemia condition is simulated, and results in Fig. 6G and fig. S20D demonstrate that the 3D epicardial bioelectronic robot successfully detects irregularities in cardiac contractility. During the hypoxia condition, the heart rate increases to receive more oxygen-rich blood, and the cardiac output increases in return. To increase the heart rate and cardiac output, all sensors experience a faster cycle with a larger amplitude due to a greater level of experienced strain. Furthermore, we simulate an ST-elevation myocardial ischemia attack through the temporary ligation of the left coronary artery (LCA), which results in a lack of blood supply to the heart muscle and further causes abnormalities in myocardial contractility, especially in the left side of the heart because it is supplied by the LCA. Accordingly, Fig. 6H shows that the sensors C2 (LV) and C3 (LA) experience a higher increase in stimulation compared with sensors C1 (RV) and C4 (RA) when myocardial ischemia occurs, indicating that the device offers sufficient sensitivity and precision in localizing the specific site of the arterial disease and other heart-related issues. The abnormal peaks in the sensor C3 may be attributed to the changes in pressure and resultant increase of contraction force across the chambers after the initial onset of myocardial infarction. Moreover, the noise from the contraction of the left ventricle and nearby lungs may also result in abnormal troughs (*51*–*54*).

Notably, common heart imaging procedures, such as MRI and Doppler echocardiogram, take at least 30 min to capture and quantify myocardial contractility changes (*55*, *56*), which can be impractical for patients with cardiac injury. However, our device provides real-time and continuous measurements to capture an evolving image of the heart. Therefore, the 3D bioelectronic epicardial robot provides a potential clinical utility in locating dysfunctional tissue and real-time monitoring the recovery of myocardial contractility through multiple output channels after cardiac surgery.

## DISCUSSION

This work presents a controlled, deterministic microfolding strategy for the design and fabrication of a broad set of 3D microelectronic systems spanning from simple to complex configurations with length scales ranging from micrometers to centimeters, across material classes from soft polymers to plastic metals, and to brittle inorganic semiconductors, providing easy access to a broad range of freestanding 3D mesostructures. A scaling law is developed to guide the folding strategy and avoid material failure for a single folding ribbon, which can serve as building blocks for constructing a broad range of ribbon-based complex structures. More than 40 examples of freestanding 3D morphable mesostructures illustrate the key ideas of microfolding and validate the utility of computational modeling to enable inverse design. Moreover, we present the fabrication of 3D folded mesostructure–templated microelectronics with deterministic control of geometry. Functionally reconfigurable 3D microatennas with various folded states display performance (e.g., S11 parameter, gain, and radiation pattern) with negligible change suggesting opportunities in the development of miniaturized electromagnetic microdevices owning great adaptability in the scenario of limited design space and deformable carrier. In addition, we demonstrate a wearable 3D vibration sensor based on the fully folded mesostructure, which has the ability to distinguish vibrational directions, frequency, and intensity simultaneously and in real time. Through integration of a wearable multivibration sensor, the vibration activity of hand can be captured in detail, which is useful in the diagnosis and rehabilitation management of hand tremor. Furthermore, we develop a 3D morphable epicardial bioelectronic robot based on the deterministic microfolding strategy that can be enclosed in a catheter structure and travel to the heart with a minimally invasive modality of intrapericardial insertion. It is capable of strain sensing at precise 3D locations to enable holistic monitoring of cardiac contractility, and we have validated its capabilities using a mouse model with cardiac injury. Overall, the 3D integrated microelectronic architectures achieved via microfolding demonstrate high tunability and controllability and promise enormous previsouly unidentified opportunities in both fundamental and applied research. Future development on the microfolding strategy could lead to utilization of structure-dependent functionality, unconventional properties from 3D microfolded structures of brittle materials, 3D integrated systems, and wireless implantable 3D microrobots for biomedical applications.

## MATERIALS AND METHODS

### Fabrication process of 3D mesostructures using deterministic microfolding

#### The preparation of folding hosts

Fabrication of an engineered folding host began with spin casting of a thin layer of PDMS (Sylgard 182 silicone elastomer; mixing ratio of 1:10 curing at 60°C vacuum for 1 hour; 5 μm in thickness) onto a glass microscope slide. Subsequently, spin casting of a second layer of PMMA (1 μm in thickness) onto the previously formed PDMS as a sacrificial layer was performed, followed by spin casting of a layer of PLGA (1 μm in thickness) as an adhesive layer. Laser cutting of hollow structure and peeling the bilayer of PDMS/PLGA away from the glass substrate yield a thin folding host. A schematic illustration is shown in fig. S1B.

#### The preparation of 3D mesostructures of monocrystalline SiNM

Preparation of 3D mesostructures of Si began with patterning of 2D precursors with the device layer of a Si-on-insulator (SOI) wafer (thickness of device layer, Si, 200 nm) by photolithography (Karl Suss MA/BA 6) and reactive ion etching (SF6 plasma etching, Alcatel AMS 100 Deep Reactive Ion Etcher). Solution immersed in buffered oxide etch partially undercut the buried silicon dioxide (SiO_2_) layer from the exposed regions and slightly from under the edges of the patterns at their periphery. Next, spin casting and photolithography formed patterns of a photoresist (S1805, 0.5 μm in thickness) as an anchor to tether the Si structure to the underlying substrate. Immersion in hydrofluoric acid (HF) fully undercut the SiO_2_ layer. After, structures retrieval onto a slab of PDMS and transferal onto a predesigned folding host allowed controlled folding of the host base to a well-defined angle via a mechanical stage and completed the 3D assembly process. A schematic illustration is shown in fig. S1 (A and C).

#### The preparation of 3D mesostructures of SiNM/PLGA

This process involved defining 2D patterned SiNM on a SOI wafer using photolithography followed by reactive ion etching as described above. First is the retrieval of Si precursors onto a PDMS stamp and transferal onto a PLGA film (2 μm in thickness). Preparation of the PLGA film began with spin coating the solution of PLGA (5 weight % in ethyl acetate) onto a hydrophobic surface of PDMS substrate, followed by slow drying at 90°C for 10 min, facilitating the bonding with Si precursor during the transfer process. After the PLGA was completely dried and fully cured (200°C, 2 hours, vacuum), the patterns of PLGA were defined by laser cutting and then were transferred onto a predesigned folding host using a PDMS stamp. A mechanical stage allowed controlled folding of the host base to a well-defined angle and completed the 3D assembly process.

#### The preparation of 3D mesostructures of Cu, Au, and Cu-coated and Au-coated PI

The preparation began with obtaining the thin films of targeted materials (Cu or Au, thicknesses range from 200 nm to 1 μm) using a sputter deposition system and placing them onto a sacrificial layer of Si wafer coated with SiO_2_ (300 nm in thickness). Photolithograph and wet etching defined patterns of the 2D precursors. Immersion in HF for 4 hours fully undercut the sacrificial layer and allowed retrieval of the 2D precursors using a PDMS stamp. Preparation of the 2D precursors of Cu- or Au-coated PI films relied on direct deposition of Cu or Au onto a thin film of PI (10 μm in thickness). Then, transfer printing of the 2D precursors onto a folding host with alignment prepared them for microfolding assembly. A mechanical stage allowed controlled folding of the host base to a well-defined angle and completed the 3D assembly process.

### Static FEA for various 3D mesostructures

3D FEAs in commercial software Abaqus were used to predict the microfolding process of 2D precursors with different patterns, dimensions, and materials, so as to guide the microstructural designs for the deterministic microfolding strategy and to establish the scaling law for predicting strain level in the mesostructures (*14*, *57*–*59*). Four-node shell elements (S4R) with second-order precision and enhanced hourglass control were used to simulate the thin mesostructures. Convergence test of the mesh size had been performed to ensure accuracy. The elastic modulus (*E*) and Poisson’s ratio (υ) used in the simulations were as follows: *E*_Si_ = 179 GPa and υ_Si_ = 0.28 for Si, *E*_Cu_ = 119 GPa and υ_Cu_ = 0.33 for Cu, and *E*_Au_ = 79.5 GPa and υ_Au_ = 0.42 for Au; and *E*_PLGA_ *=* 4.5 GPa and υ_PLGA_ = 0.34 for PLGA, *E*_PI_ *=* 2.5 GPa and υ_PI_ = 0.34 for PI, and *E*_Parylene_ *=* 4.5 GPa and υ_Paralene_ = 0.4 for parylene. Because the brittle fracture is more likely to occur in the Si mesostructures, the ɛ_max_ was obtained by FEA simulation to determine whether the microfolded 3D structures will fracture. For the mesostructures made of metallic materials (Cu and Au), we have a concern about whether they will fail because of yielding. The FEA results of microfolded 3D metallic material mesostructures show their deformations with the equivalent strain based on Von Mises Stress, denoted by ɛ_VMS_.

### Fabrication and measurement of 3D reconfigurable microantennas and electromagnetic simulations

#### Fabrication of microantenna

The process began with deposition of Cu nanomembrane (thickness ~110 nm) onto a PI film (thickness ~10 μm) by magnetron sputtering. Laser cutting the film of Cu/PI bilayer formed the planar dipole microantenna with the parameterization, as shown in fig. S9A. Deterministic folding of the planar antenna on a predefined folding host to a well-defined angle (Fig. 4B) enables precise control of the 3D transformation. The as-prepared 3D mesostructures with different folding angles are maintained via strategically shaping the PDMS substrate as shown in fig. S1D.

#### Microantenna measurement

The S-parameters were tested by an Agilent Technologies E5071C network analyzer (300 kHz to 20 GHz) to measure the resonant frequency and reflection coefficient. The antenna was connected with the network analyzer via SMA (SubMiniature version A) connector (fig. S9C).

#### Electromagnetic simulation

The electromagnetic modeling was carried out by using the software Ansys HFSS to obtain the reflection coefficient (S11), port impedance (Z11), and gain of the microdipole antennas with various degrees of microfolding for guiding the 3D miniaturization of the antenna. The 3D folded antenna models, obtained from the Abaqus mechanical simulation, were imported into the Ansys HFSS; then, the geometric model was processed through thickening and Boolean operations. To make the simulation closer to the real experimental situation, the SMA used in the experiment and the Cu wire connected between the SMA connector and the antenna were added into the electromagnetic model. The default adaptive convergence condition, together with a spherical surface of 40 mm in radius as the radiation boundary, guaranteed computational accuracy. The electrical conductivity of the Cu film (thickness ~110 nm) was around 2.6 × 10^6^ S/m based on DC resistance measurement, considering the effects of the thin-film confinement. Other material properties use the default values included in the Ansys HFSS material library.

### Fabrication and measurement of 3D vibration sensor and dynamic FEA

#### 3D vibration sensor preparation

The fabrication began with phosphorous-doping the top Si layer (thickness, 200 nm) onto the SOI wafer (doping concentration, ~2 × 10^20^/cm^3^). Next, undercut etching of the buried oxide layer was followed by transfer printing the Si membrane with a serpentine shape (fig. S13A) onto a film of parylene (thickness, ~10 μm). Plasma-enhanced chemical vapor deposition (Kurt Lesker PVD 75) and a predesigned hard mask defined patterns of metal (Cr/Au; thicknesses, 10 and 150 nm) for contacts to the ends of the serpentine. An additional layer of parylene formed an electrically insulating encapsulation layer with openings aligned to the metal contacts. Cutting through this layer and the base layer using a laser beam defined the 2D precursor (fig. S13B). Then, bonding the precursor with a proof mass (PDMS/Cu composite; length by width by height, 1 mm by 1 mm by 1 mm; density, 4.5 g/cm^3^) and transferring it onto a predesigned folding host prepared the system for 3D transformation. A mechanical stage allowed controlled folding of the host base to a well-defined angle and completed the 3D assembly process. A PDMS holding substrate provides structural support to lock the 3D vibration sensor in a partially folded state (fig. S1D).

#### 3D vibration sensor measurement

All experiments were performed in accordance with institutional guidelines and regulations. The experimental protocol (protocol number 22-0163) was approved by the office of Human Research Ethics at the University of North Carolina. The data acquisition system included a linear actuator (fig. S13D) and a PowerLab computer interface (Model 16/35, AD Instruments). The linear actuator produced periodic vibrations with programmable frequencies between 0 and 5 Hz. The PowerLab system allowed recordings of output voltage.

#### Dynamic FEA for 3D vibration sensor

To guide the design of 3D vibration sensor and determine the relationship between the strain change of the 3D structure and vibrational frequency, the modal analysis and steady-state dynamics analysis were performed in commercial software Abaqus. First, the subspace iteration method in modal analysis was adopted to find the structural fundamental frequency and the responding vibrational form. Second, the steady-state dynamic analysis was used to determine the relationship between strain change of the 3D structure and vibrational frequency and further guide the location of the sensor patch. The bottom of the 3D vibration sensor was excited by a harmonic displacement with an amplitude of 15 mm along each coordinate axis, and the frequency sweep analysis of the 3D vibration sensor was performed. The 3D vibration sensor model was imported from static FEA results. Eight-node hexahedron elements (C3D8R) were adopted for the proof mass (Cu/PDMS composite), and S4R were adopted for the thin ribbon of Si/PLGA bilayer with the section of composite layup in Abaqus. The lower surface of the proof mass was tied to the upper surface of the composite layer. The elastic modulus (*E*), Poisson’s ratio (υ), and density (ρ) in the simulations were *E*_Si_ = 179 GPa, υ_Si_ = 0.28, and ρ_Si_ = 2.33 g/cm^3^ for Si; *E*_PLGA_ *=* 4.5 GPa, υ_PLGA_ = 0.34, and ρ_PLGA_ = 1.38 g/cm^3^ for PLGA; and *E* = 1.2 GPa, υ = 0.48, and ρ = 4.5 g/cm^3^ for the proof mass.

### Fabrication of transformable epicardial bioelectronic robot and in vivo animal test

#### 3D epicardial bioelectronic robot preparation

The Au nanomembrane (thickness, ~50 nm) was deposited by magnetron sputtering on the PI film (thickness ~10 μm). The planar epicardial bioelectronic patch with the parameterization shown in fig. S18 was formed using the laser cutting machine. The 3D epicardial bioelectronic robot with strain sensors was achieved by folding the planar patch on a predefined folding host. Last, the as-prepared 3D robot was attached to a catheter as shown in fig. S19.

#### In vivo animal experiment

Procedures used in this study were reviewed and approved by the Institutional Animal Care and Use Committee and Research Animal Resources at the North Carolina University Chapel Hill (ID 21-241.0). Female mice (weight, 20 to 30 g; age, 10 weeks) were purchased from the Jackson Laboratory. Note S3 describes the detailed surgery process. Thoracotomy on the animal opened a small window for the probe to be placed on the cardiac surface, which is followed by data collection. The electrocardiography and heart rate were monitored simultaneously using commercial equipment (PowerLab). The hypoxia and ischemia condition tests were conducted by adjusting the tidal volume on the ventilator and temporarily occluding the LCA, respectively.

## Supplementary Materials

### This PDF file includes:

Notes S1 to S3 Figs. S1 to S20 References
