# Supplementary material for: 3D morphable systems via deterministic microfolding for vibrational sensing, robotic implants, and reconfigurable telecommunication
Source: Sci Adv. 2022 Dec 21;8(51):eade0838. doi: 10.1126/sciadv.ade0838 (PMC9770994; doi:10.1126/sciadv.ade0838)
Supplement: Supplementary file 1 — Notes S1 to S3 Figs. S1 to S20 References [file sciadv.ade0838_sm.pdf]

Science Advances
AAAS

Supplementary Materials for

3D morphable systems via deterministic microfolding for vibrational sensing, robotic implants, and reconfigurable telecommunication

Lin Zhang *et al.*

Corresponding author: Zhaoqian Xie, zxie@dlut.edu.cn; Wubin Bai, wbai@unc.edu

Sci. Adv. **8**, eade0838 (2022)
DOI: 10.1126/sciadv.ade0838

The PDF file includes:

Notes S1 to S3
Figs. S1 to S20
Legends for movie S1
References

Other Supplementary Material for this manuscript includes the following:

Movie S1

Supplementary Note 1: Deriving the scaling law of ε_{max} in folding a slender ribbon

Derivation of the scaling law (as shown in S1) was based on the FEA of the microfolding process of a single slender ribbon, which can serve as a guiding principle for designing complex ribbon-based patterns (e.g., **Fig. 1I** and **Fig. S7Ai**). The scaling law focuses on four geometric parameters of a ribbon and its relationship with a folding host: length *l*, width *w* and thickness *t* of the ribbon, as well as the staggered distance *d* between two bonding sites (as illustrated in **Fig. 1A**). For a ribbon undergoing a bending process, the ε_{max} is linearly proportional to κt, where the curvature κ is proportional to l^{-1}. The FEA results verified that the influence of the non-dimensional ratio *w/l* on ε_{max} can be ignored for *w/l* < 0.1 (**Fig. S5E**). Then, the ε_{max} can be expressed as

$$\varepsilon_{max} = \frac{t}{l} * \varphi\left(\frac{d}{l}\right) \qquad \text{(S1)}$$

where φ was a function of *d/l*. For the current study, the range of *d/l* from 0 to 0.2 was considered in the scaling law. The straight lines in **Fig. S5B** to **D** clearly verified the above scaling law, and their slopes gave the value of the function φ under the corresponding variable *d/l*. Treating *d/l* as an independent variable and $\varepsilon_{max} \cdot l/t$ as function value (**Fig. S5E**) enables the determination of the function φ by fitting the FEA results. Finally, the ε_{max} was given by

$$\varepsilon_{max} = \frac{t}{l} * \left(-30.3\left(\frac{d}{l}\right)^2 + 5.65\right)$$

(S2)

Supplementary Note 2: The S11 parameter of 3D reconfigurable microantennas

The reflection coefficient S11 describes amplitude of the power reflected from the antenna that is caused by an impedance discontinuity in the transmission medium.[61] In a broad sense, it can be determined by the difference between input and out impedance based on the following formula[62]

$$S11 = 20\, log_{10}\left|\frac{Z11 - Z0}{Z11 + Z0}\right|$$

(S3)

In the equation, Z11 is the input impedance of the antenna port, and Z0 is the internal impedance of the antenna excitation. It is obvious that the more perfect the impedance match is, the smaller S11 value will be.

Supplementary Note 3: In vivo animal test of transformable epicardial bioelectronic robot

The animals were kept on a 12-hour light-dark cycle in a temperature-controlled room. The animal was initially placed under anesthesia in a chamber with isoflurane gas (5% isoflurane and 100% oxygen). Once consciousness was lost, the animals were intubated with a 16-gauge flexible catheter, and endotracheal tube was connected to a mechanical ventilator (Braintree VentStar ventilator) that provided positive-pressure ventilation with oxygen/isoflurane. The ventilator was set based on animal weight: tidal volume (Vt, milliliters) = $6.2 \times M^{1.01}$ and respiration rate (RR min^{-1}) = $53.5 \times M^{-0.26}$, M = animal weight in kilograms. Each mouse was connected to a vaporizer that delivers approximately 2.0% isoflurane driven by 100% oxygen. Mice were placed in the dorsal decubitus position on a warming platform. Intradermal bupivacaine was infiltrated at the incision sites approximately 10 min before incisions. Mice were maintained at approximately 37°C on a heating pad, with body temperature monitored throughout the experiment using a rectal temperature probe. The animal's hair was removed from the surgical site with hair removal cream NAIR after shaving. The surgical areas were scrubbed and disinfected with a povidone iodine prep pad, and the area was then wiped with an alcohol prep pad. Peripheral blood oxygen saturation was monitored throughout the experiment using a commercial pulse oximetry system (MouseSTAT Jr., Kent Scientific Co.).

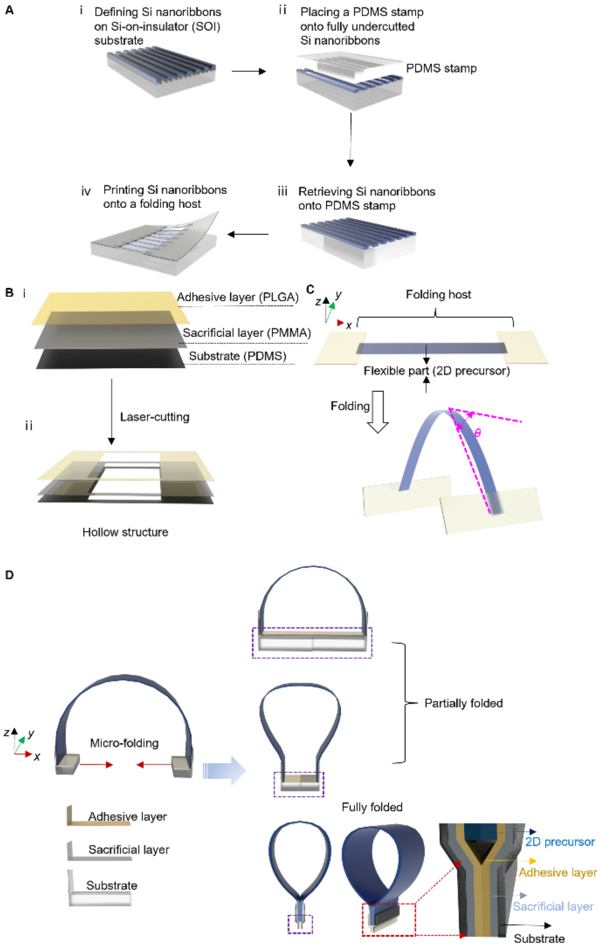

Fig. S1. Microfolding assembly of 3D microstructures of monocrystalline Si. (**A**) Schematic illustration showing the transfer printing process to produce nanoribbons of monocrystalline silicon (Si): (i) Lithographically defining Si nanoribbons on a silicon-on-insulator (SOI) substrate. (ii) Placing a polydimethylsiloxane (PDMS) stamp onto fully undercut Si nanoribbons. (iii) Retrieval of Si nanoribbons from the SOI substrate onto a PDMS stamp. (iv) Transferal of Si nanoribbons from the PDMS stamp onto a folding host. (**B**) Schematic illustration showing the fabrication process of a multilayer of folding host: (i) Spin coat poly(methyl methacrylate) (PMMA) and poly(lactic-co-glycolic acid) (PLGA) solution onto a PDMS sheet as the sacrificial layer and adhesive layer, respectively. (ii) Laser cut an outline on the PLGA/PMMA/PDMS hybrid sheet to form a well-defined trench. Here, the multilayer design of folding host consisting of the substrate (e.g., PDMS) with controllable dimension, a sacrificial layer (e.g., PMMA), and an adhesive layer (e.g., PLGA) to enable the fixation of partially folded mesostructure. (**C**) Schematic illustration of a folding structure composed of a folding host (PLGA/PMMA/PDMS sheet) and the patterned 2D precursor. The folding angle θ is defined as the inclination angle of the folding host. (**D**) Schematic illustration showing a fixation mechanism to lock 3D folded mesostructures at various folding angles. Here, we adopt the multilayer design of folding host that can be either shaped or removed on demand.

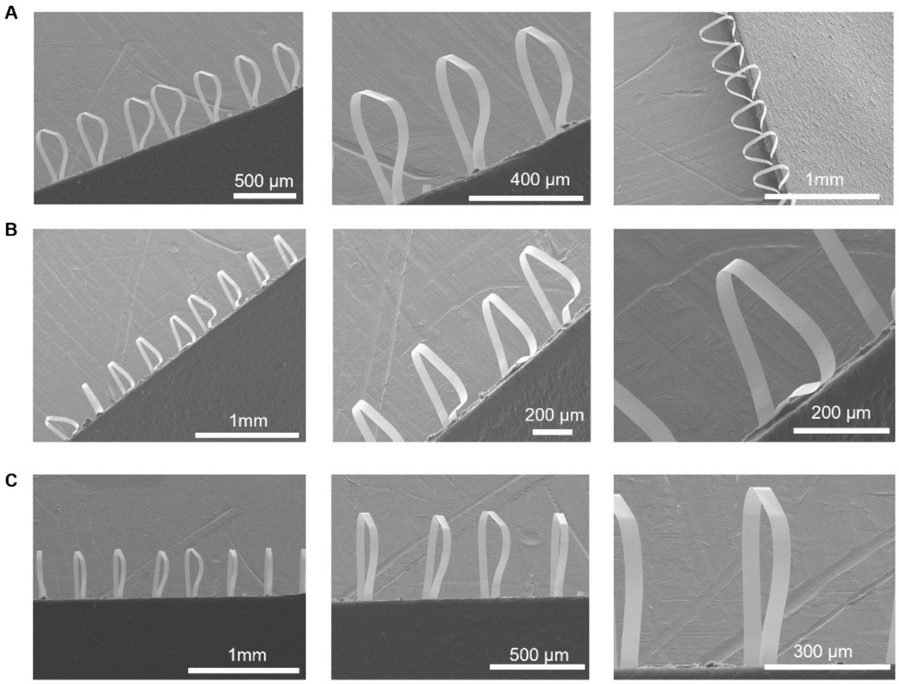

Fig. S2. SEM images of 3D Si hoops achieved by deterministic microfolding. The images display the 3D Si hoops under different projections of view and with various magnifications. (**A**) side-view; (**B**) top-view; and (**C**) front-view. Here, the geometric parameters *l* and *d* are 1500 μm and 0 μm, respectively. The folding angle θ is 90°.

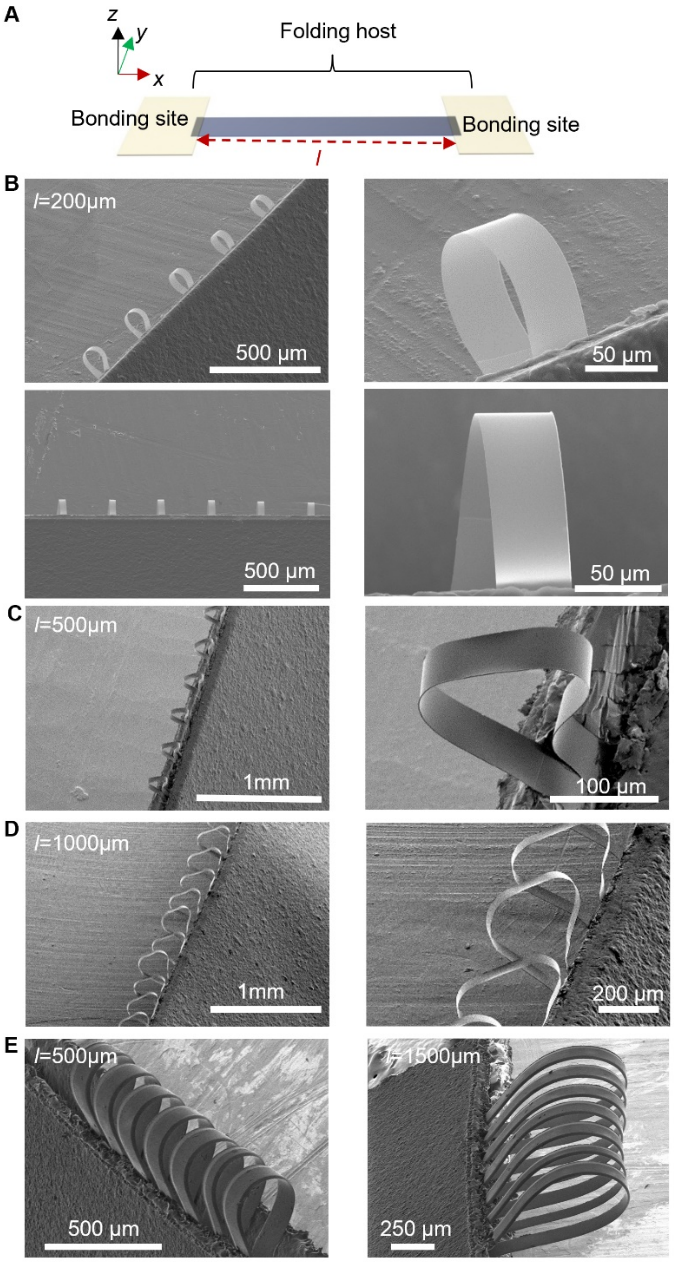

Fig. S3. Microfolded 3D Si hoops with various radii (*R*) of curvature. (**A**) Schematic illustration indicating the definition of the design parameter *l* in ribbon-shape 2D precursors. (**B** to **D**) SEM images showing the 3D Si hoops with various radii of curvature achieved by tuning the *l*. Here, it is observed that the *R* increases as the *l* increases (from 200 μm to 1500 μm). (**B**) *l* is 200 μm. (**C**) *l* is 500 μm. (**D**) *l* is 1000 μm. The residual photoresists on the edges of the Si nanoribbons in (**B**) are removed using oxygen-plasma etching and effectively prevents them from bending downward. (**E**) SEM images of 3D hoops from 2D filamentary serpentine ribbons of polyimide (PI) bonded at the two ends to a folding host. Left: *l* is 500 μm. Right: *l* is 1500 μm.

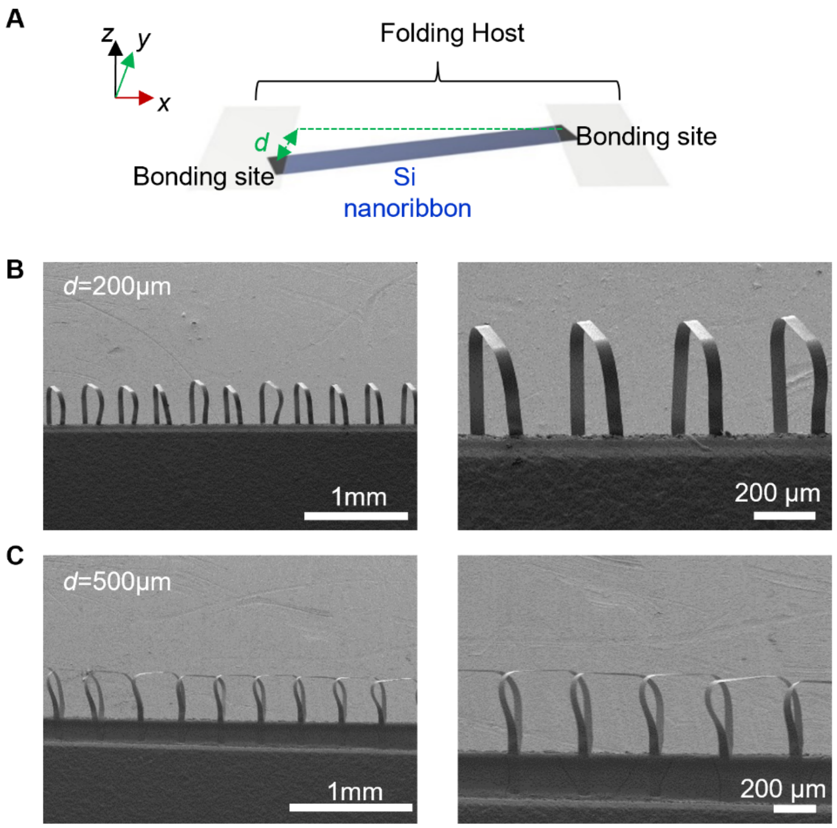

Fig. S4. Microfolded 3D Si hoops with twisting architecture. (**A**) Schematic illustration indicating the implementation of the design parameter *d* in ribbon-shape 2D precursors. (**B** and **C**) Experimental SEM images demonstrating the precise control of orientation of the 3D hoops via tuning the parameter *d*. Here, the *d* values are 200 μm and 500 μm for the inverted pyramids (**B**) and side-by-side archways (**C**), respectively.

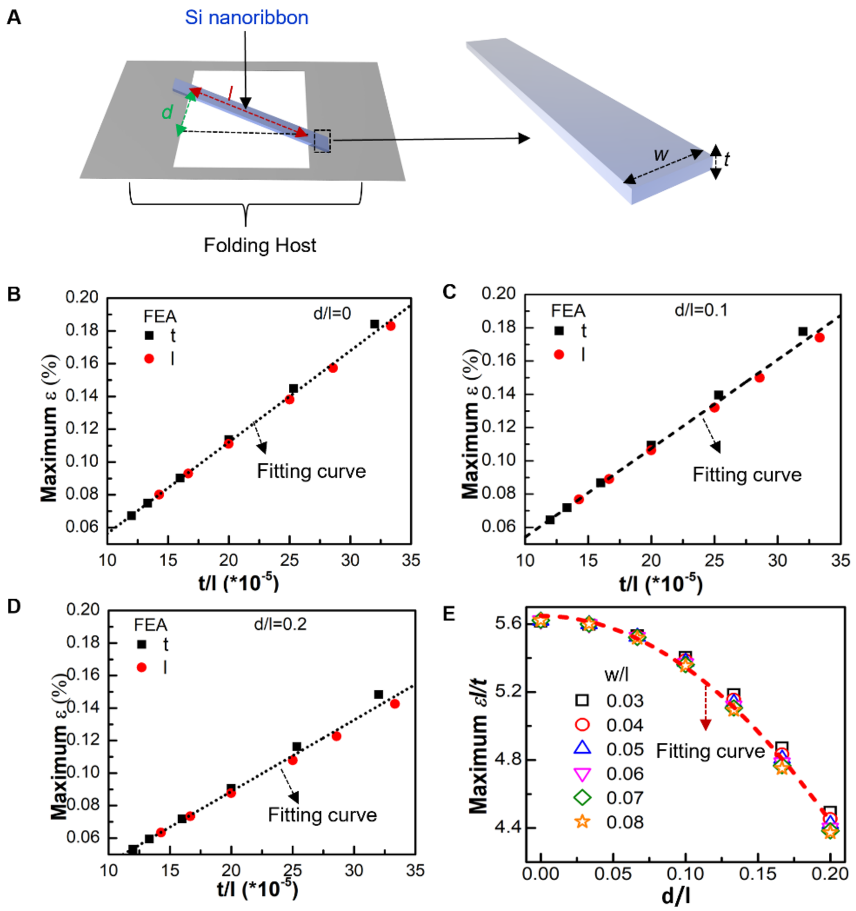

Fig. S5. Computational modeling on the influence of various geometrical parameters on maximum principal strain (ε_{max}) in 3D folded hoops. (**A**) Illustration of the hinge structure consisting of the folding host and 2D precursor of Si ribbon. The length, width, and thickness of the individual ribbon are labeled as *l*, *w,* and *t*, respectively. The distance between two bonding sites along *y*-axis is labeled as *d*. (**B** to **D**) The maximum principal strain ε_{max} shows a linear relationship with dimensionless parameter *t/l* through FEA simulation when *d/l* =0 (**B**), 0.1 (**C**) and 0.2 (**D**). (**E**) The fitting for dimensionless parameter *εl/t* and *d/l* with different *w/l* values ranging from 0.03 to 0.08. Here, the *εl/t* decreases with increasing *d/l,* depending weakly on *w/l.*

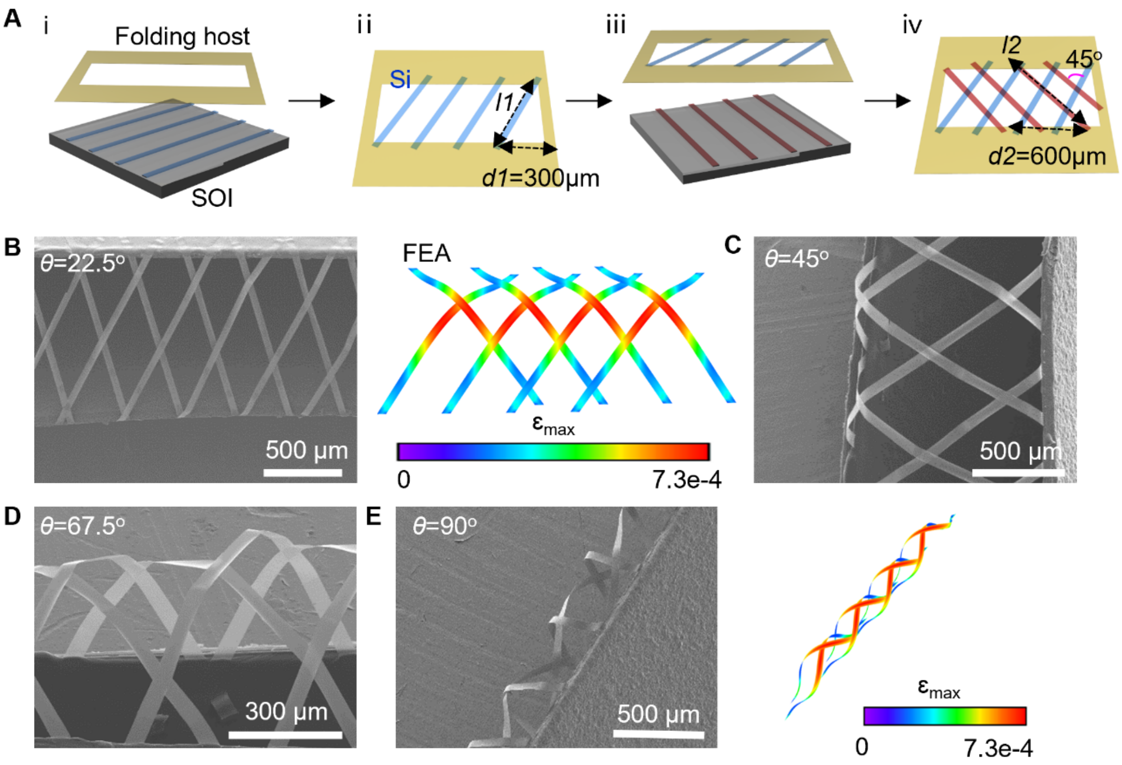

Fig. S6. Micro folded 3D double helix via folding 2 layers of 2D filamentary nanoribbons of Si. (**A**) Schematic illustration showing the route for forming a bilayer stack of Si nanoribbons with a mesh pattern: (i) and (ii) Transferring Si nanoribbons with a PDMS stamp onto a trenched PLGA/PDMS folding host, in which the design parameters l_1 and d_1 of the hinge structure are 1500 μm and 300 μm, respectively. (iii) and (iv) Bonding another layer of Si nanoribbons onto the same folding host with registration. For the second layer of Si nanoribbons, the parameter l_2 and d_2 are 1500 μm and 600 μm, respectively. The angle between the two 2D precursor layers is fixed at ~45°. The top and bottom layers of Si nanoribbons are colorized as blue and red, respectively. (**B** to **E**) SEM images and corresponding FEA results of transitional states of the 3D Si double helix structures via controlling the folding angle of the bilayer 2D precursors of Si nanoribbons. θ = 22.5° in (**B**), 45° in (**C**), 67.5° in (**D**), and 90° in (**E**).

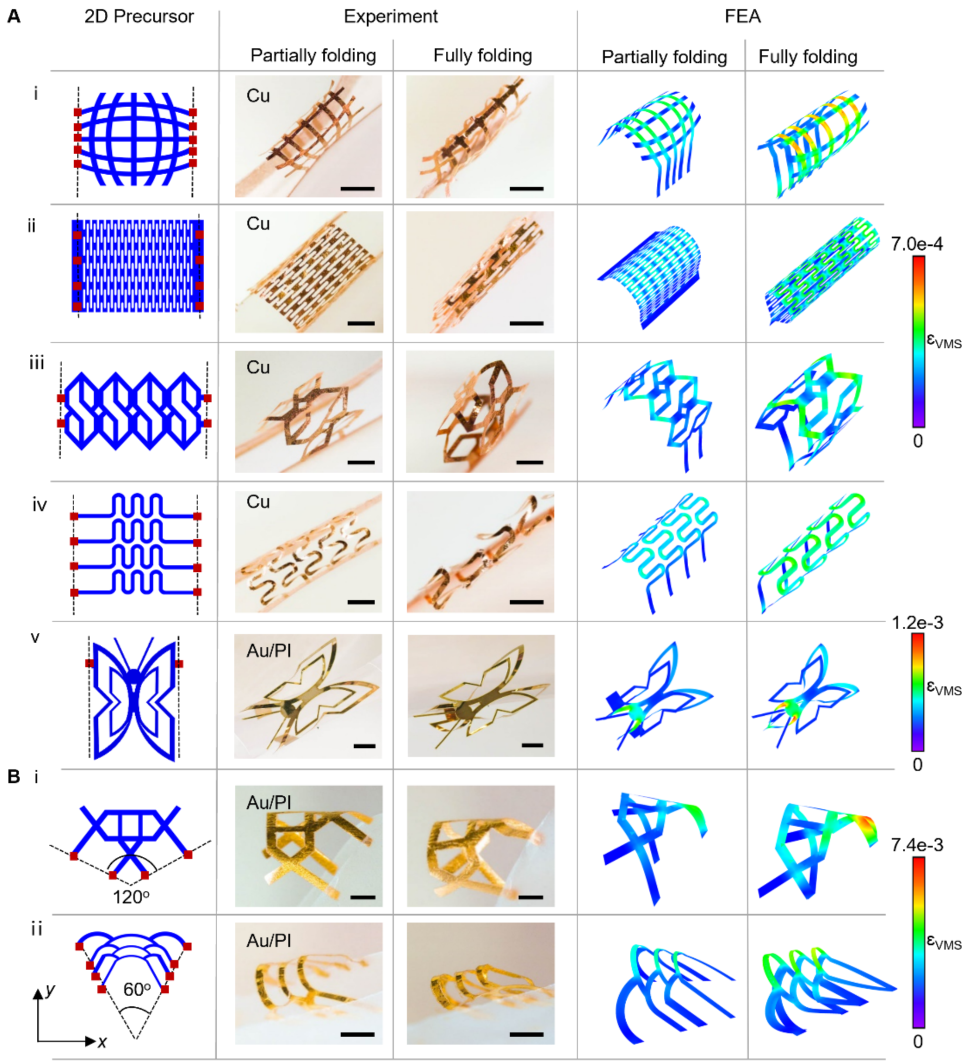

Fig. S7. Representative 3D morphable mesostructures based on microfolding assembly. (**A** and **B**) 2D geometries, experimental images, and FEA results of various 3D mesostructures via parallel folding registration (**A**) and angled folding registration (**B**). The fishing net (**A**i), sliding door (**A**ii), hollow-out lampshade (**A**iii) and an array of hair hoops (**A**iv) are made of Cu (thickness ~1 μm). (**A**v) A butterfly made of a bilayer of Au (thickness ~150 nm) and PI (thickness ~10 μm) consists of outer and inner wings with minimum interconnections between each other, and the bonding sites locate only on the outer wings. As microfolding initiates, the outer wings undergo large deformation as a result of mechanical constraints from the bonding sites, while there is little shape transformation exhibited on the inner wings. The eagle-like (**B**i) and insect antennae-like (**B**ii) structures are made of a bilayer of gold nanomembrane (Au, thickness ~150 nm) and polyimide (PI, thickness ~10 μm). Scale bars, 1 mm.

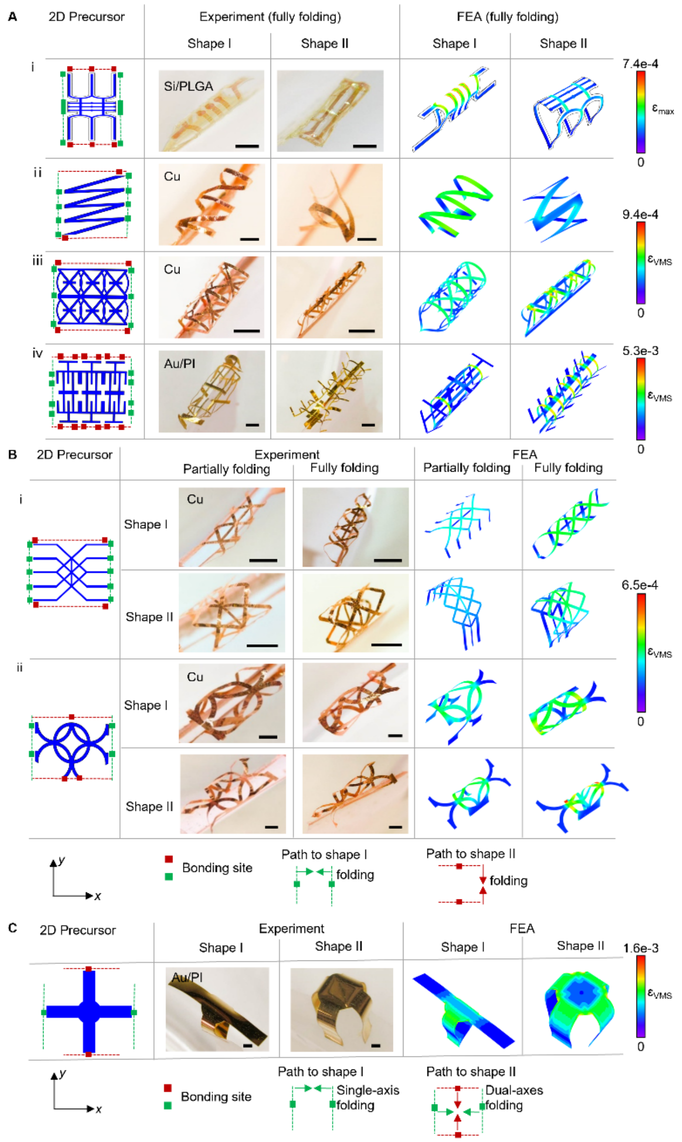

Fig. S8. Representative demonstration on 3D reconfiguration via microfolding assembly. (**A** to **C**) 2D precursors, experimental images, and corresponding FEA results of ribbon-shaped mesostructures via switching folding paths. (**A** and **B**) Here, folding paths with corresponding folding directions aligned along *y*-axis and *x*-axis lead to two distinct 3D configurations, shape I and II, respectively, from same 2D precursors. Furthermore, (**C**) two distinct configurations, a bamboo dragonfly (shape I) and four-legged footstool (shape II), come from the same 2D precursor via adopting folding path along a single axis and duo axes, respectively. (**A**i) The 3D mesostructures are made of bilayer of Si (thickness ~200 nm) and PLGA (thickness ~2 μm); (**A**ii and **A**iii) The 3D mesostructures are made of Cu (thickness ~1 μm); (**A**iv) The 3D mesostructures are made of bilayer of Au nanomembrane (thickness ~150 nm) and PI (thickness ~10 μm). The 3D mesostructures in (**B**) are all made of Cu (thickness ~1 μm). (**C**) The mesostructure is made of a bilayer of gold nanomembrane (Au, thickness ~150 nm) and polyimide (PI, thickness ~10 μm). Scale bars, (**A**i) 500 μm, (**A**ii to **A**iv) 1 mm, (**B**i and **B**ii) 1 mm, and (**c**) 1 mm.

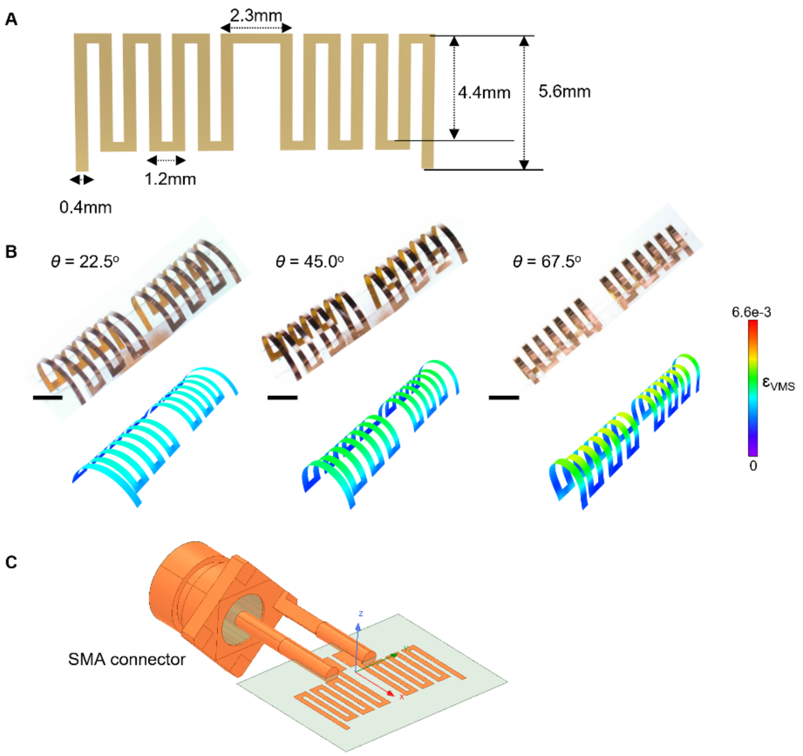

Fig. S9. 3D reconfigurable dipole microantennas. (**A**) Schematic illustration of geometrical dimensions of the 2D antenna with a zigzag structure constructed with a bilayer of Cu nanomembrane (110 nm) and PI (10 μm). (**B**) The optical images and FEA results of the 3D folded antennas with different folding angles θ (left θ=22.5°, middle θ=45°, right θ=67.5°). (**C**) An antenna was connected to a coaxial cable by a SMA connector. Scale bars, 2mm.

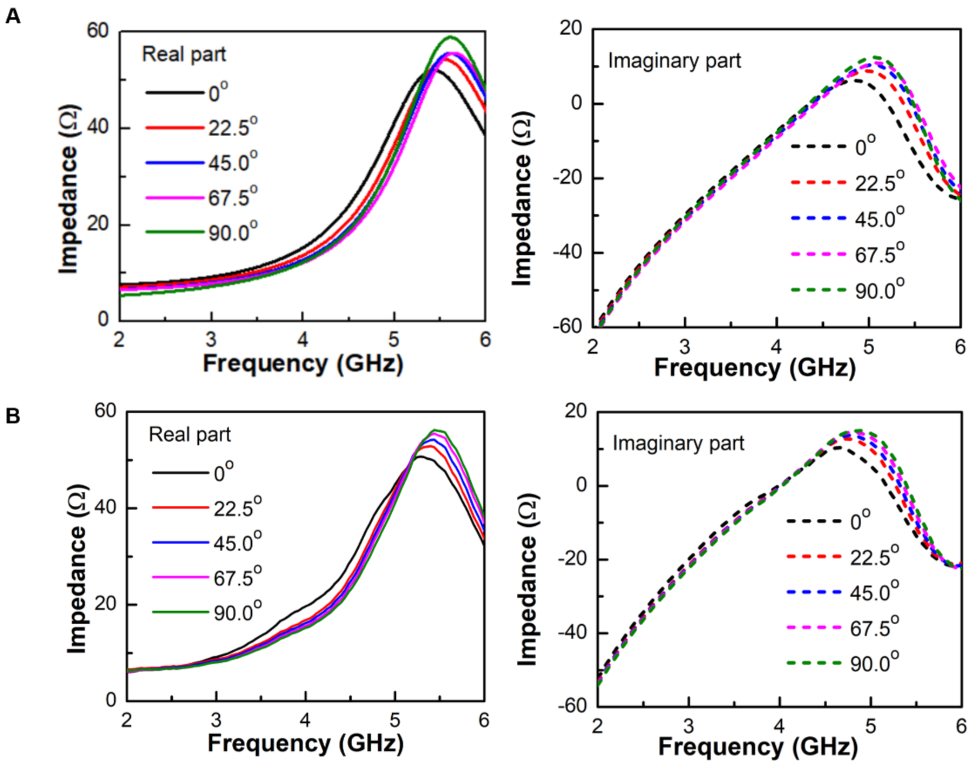

Fig. S10. Measured impedance of microantennas as a function of frequency. (**A**) Simulated and (**B**) measured impedance versus frequency response for antennas with different folding angles.

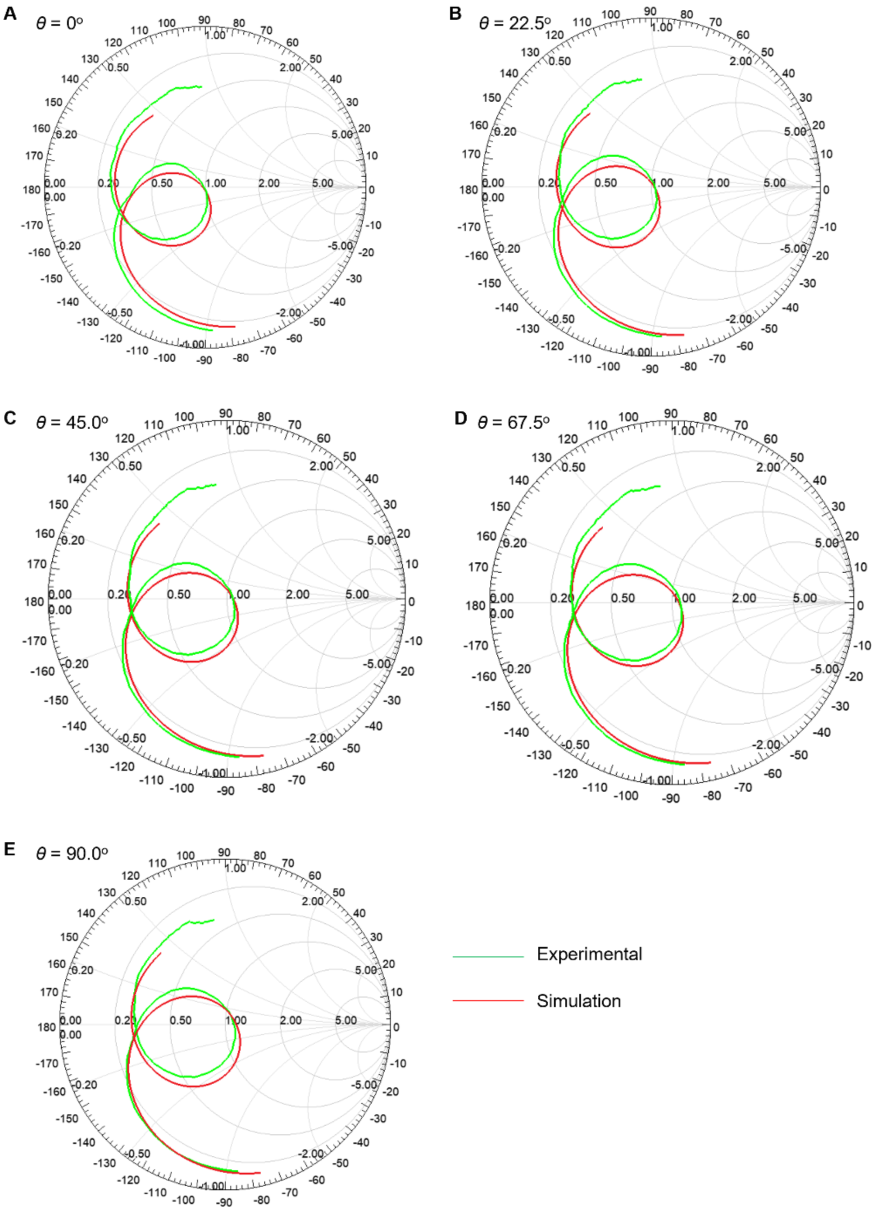

Fig. S11. The smith chart of the measured and simulated input impedance for antennas with different folding angles. (**A**) θ=0°; (**B**) θ=22.5°; (**C**) θ=45°; (**D**) θ=67.5°; (**E**) θ=90°.

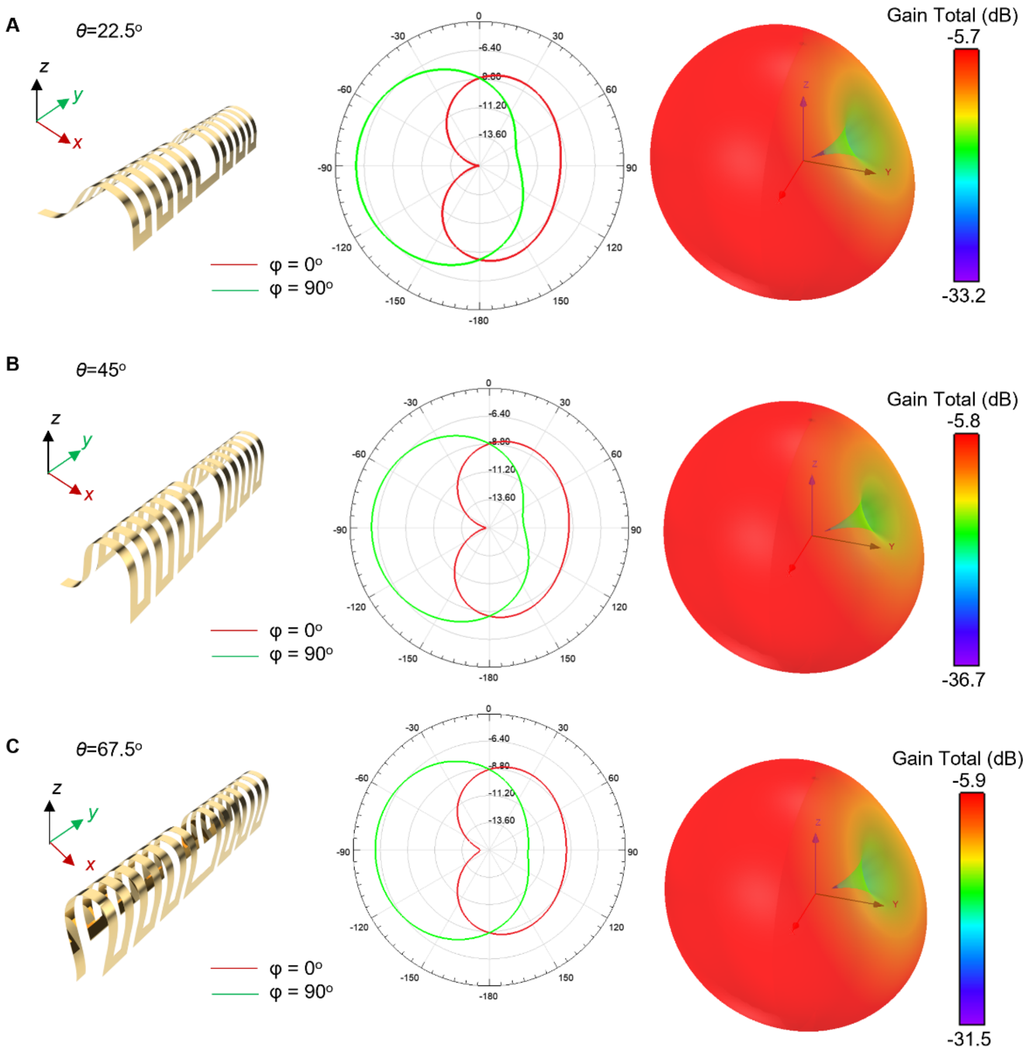

Fig. S12. The simulated 2D- (middle column) and 3D- (right column) radiation patterns of 3D microatennas with various folding angles *θ*. (**A**) θ=22.5°; (**B**) θ=45°; (**C**) θ=67.5°.

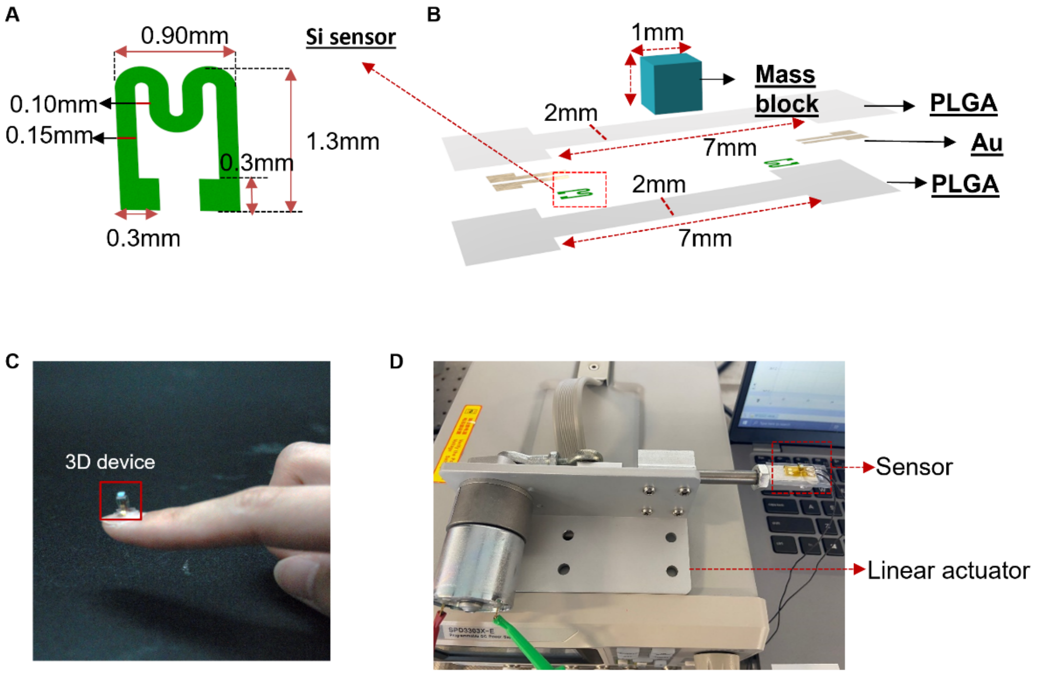

Fig. S13. Materials and construction of a 3D vibration sensor. (**A**) Schematic illustration of a SiNM sensor with the serpentine structure. (**B**) Schematic illustration of a 3D vibration sensor that integrates SiNM sensor, gold interconnects, and a proof mass, all on a thin parylene film. (**C**) Optical image of a wearable 3D vibration sensor attached on a fingernail. (**D**) The optical image of a 3D vibration sensor fixed on a linear actuator that can produce periodic vibrations with programmable frequencies between 0 Hz and 5 Hz.

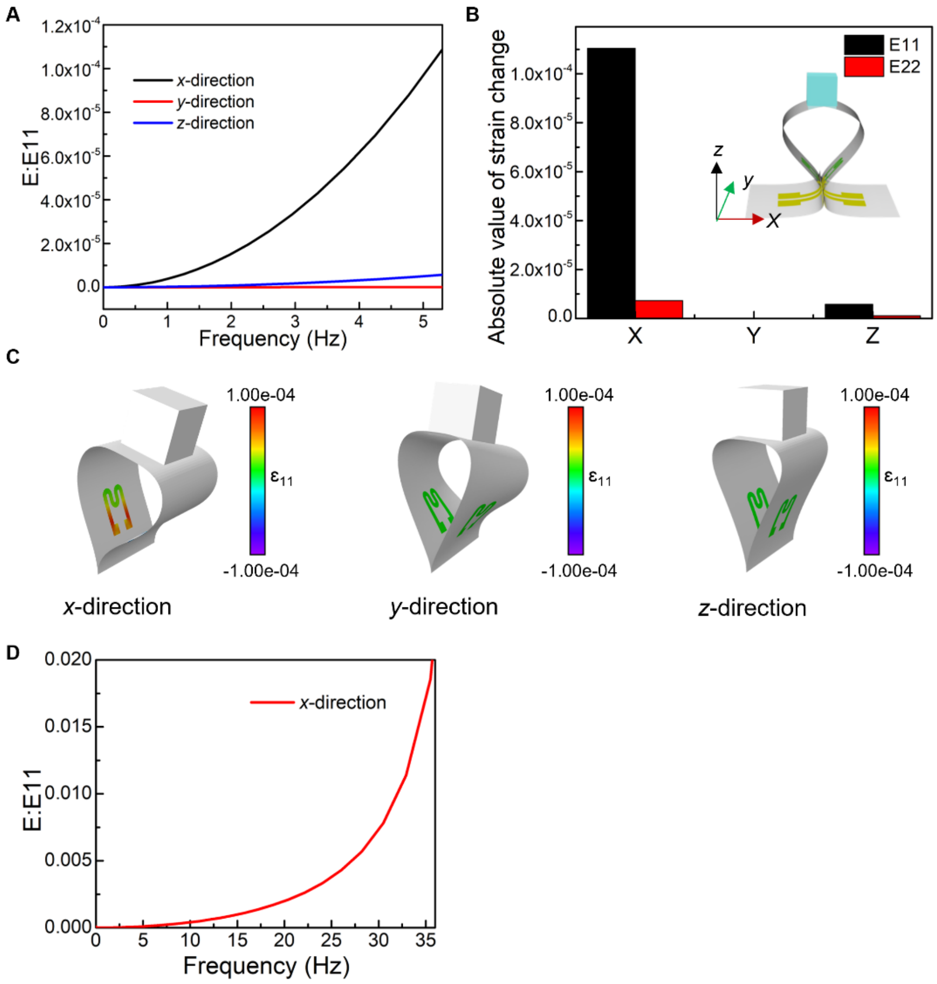

Fig. S14. A wearable vibration sensor based on a 3D microfolded mesostructure. (**A** and **B**) The FEA analysis on the relative strain change of the SiNM sensor as a function of vibrational frequency along 3 orthogonal directions. Here, computational analysis reveals that the vibration in the *x*-direction plays a dominant role in the strain change of the silicon sensor in the frequency range from 0-5.3 Hz covering medical relevance of the hand rest tremor,[45,46] which provides theoretical guidance for the design and placement direction of the 3D vibration sensor. (**C**) The simulated distribution of strain at a vibrational frequency 3.3 Hz. (**D**) The FEA analysis on the relative strain change of the Si sensor as a function of vibrational frequency along *x*-direction. The range of vibrational frequency is 0-36 Hz. Here, E11 and E22 represent projected components of the total strain of the Si sensor along two mutually perpendicular directions associated with the length and width of the PLGA ribbon, respectively.

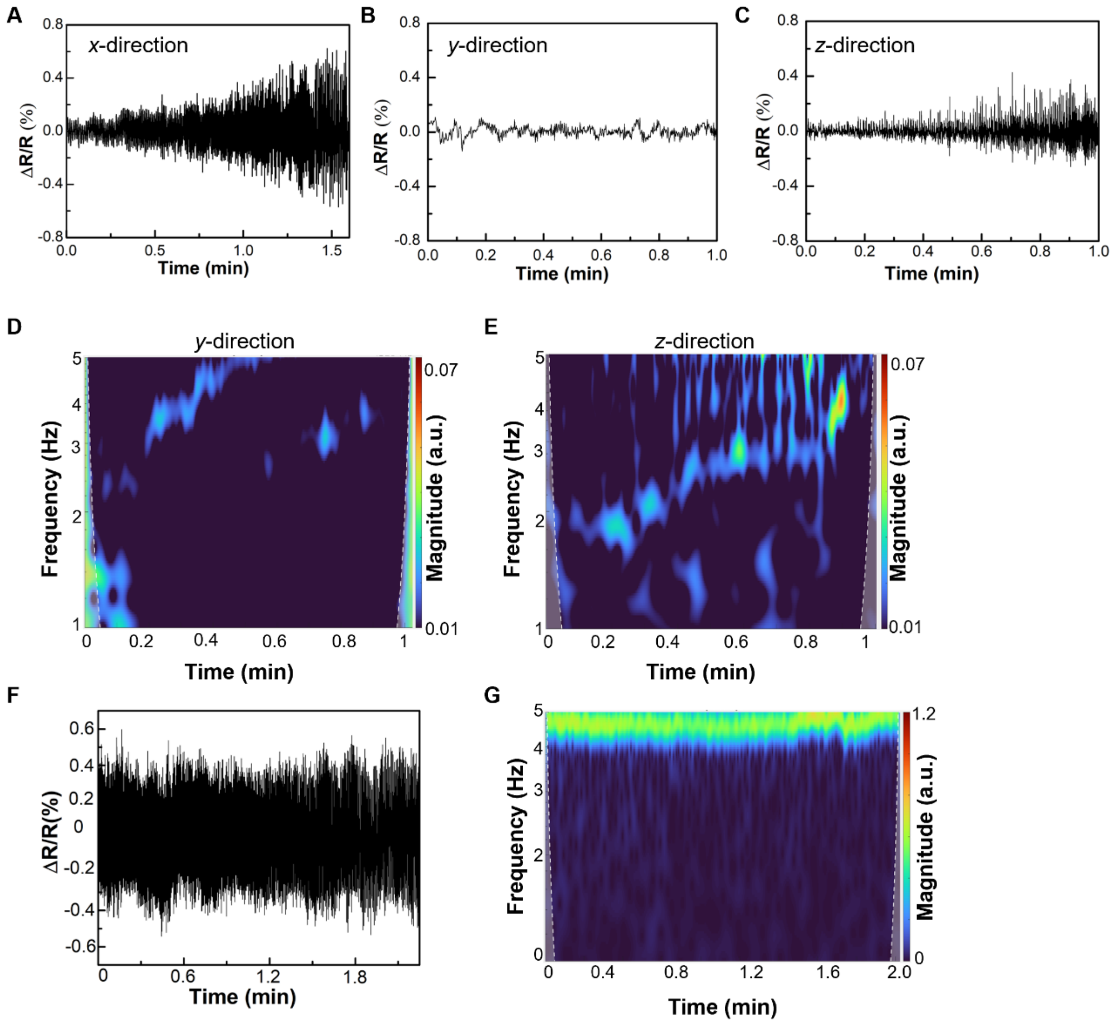

Fig. S15. Representative measurements of a 3D vibration sensor based on a fully folded mesostructure. (**A** to **C**) Representative time-domain resistive change of the wearable 3D vibration sensor in response to vibrational activity in 3 orthogonal directions. The vibrational activity is realized by a linear actuator at various vibrational frequencies ranging from 1 Hz to 5 Hz. (**A**) *x*-direction, (**B**) *y*-direction and (**C**) *z*-direction. (**D** and **E**) The corresponding frequency spectra based on (**B**) and (**C**), respectively. (**F** and **G**) Representative change of resistance in the time domain (**F**) and corresponding frequency spectrum (**G**) of vibrational measurement from a 3D vibration sensor in response to a 5-Hz vibration along *x*-direction after being operated for 50000 cycles (each cycle consists of 10 mins of cycling vibrations (frequency 5 Hz and *x*-direction).

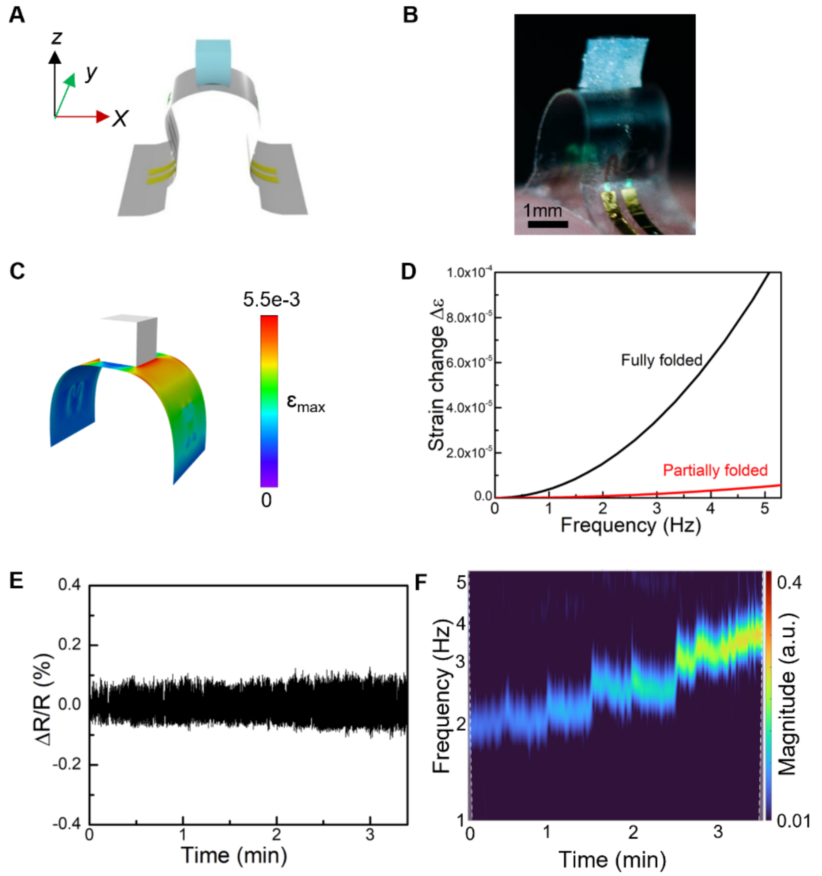

Fig. S16. Representative measurements of a 3D vibration sensor based on a partially folded mesostructure. (**A**) Schematic illustration and (**B**) optical image of a 3D vibration sensor based on a partially folded mesostructure and its local coordinate system. (**C**) FEA results of the 3D partially-folded mesostructure used by the wearable 3D vibration sensor. (**D**) The comparison of the FEA analysis, between a fully-folded and a partially folded mesostructures, on the relative strain change of the SiNM strain sensor as a function of vibrational frequency along *x* direction with vibrational frequency ranging from 0 to 5.3 Hz. (**E**) Representative time-domain resistive change measured by the 3D vibration sensor in response to vibrational activity in *x*-direction at various vibrational frequencies ranging from 1 Hz to 5 Hz. (**F**) The corresponding frequency spectrum based on (**E**).

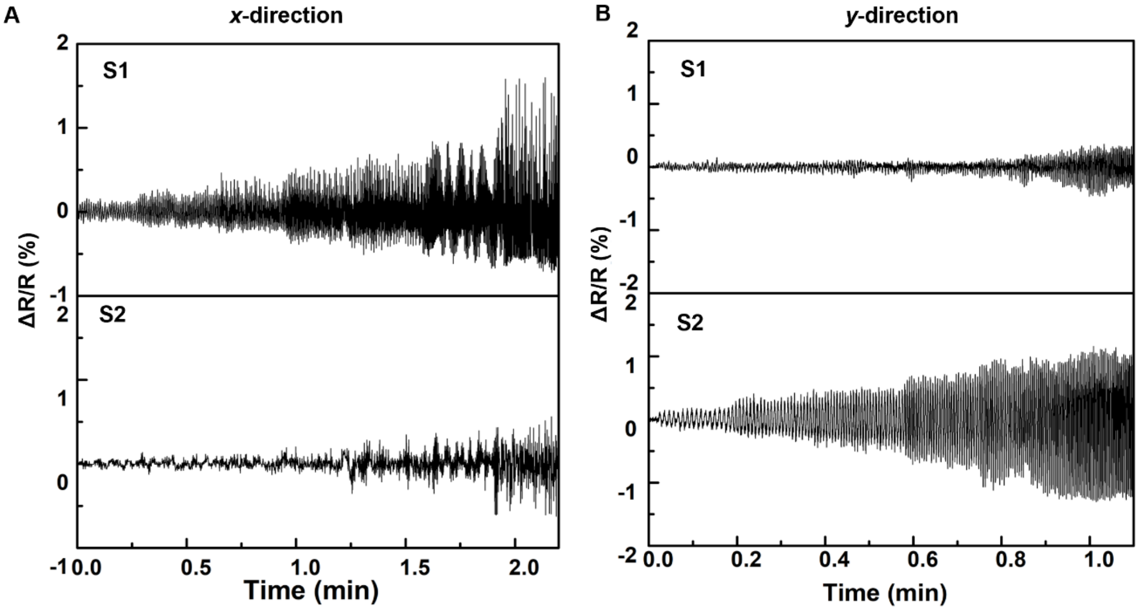

Fig. S17. Representative time-domain resistive change measured by the 3D vibration sensor in response to finger vibrations with different directions. (**A**) Vibration direction along *x*-axis. (**B**) vibration direction along *y*-axis (as defined in **Fig. 5F**). Here, the data are collected from an integrated pair of vibration sensors (labeled as S1 and S2, respectively, as shown in **Fig. 5F**), which compose a vector data that defines directionality of vibrational activities.

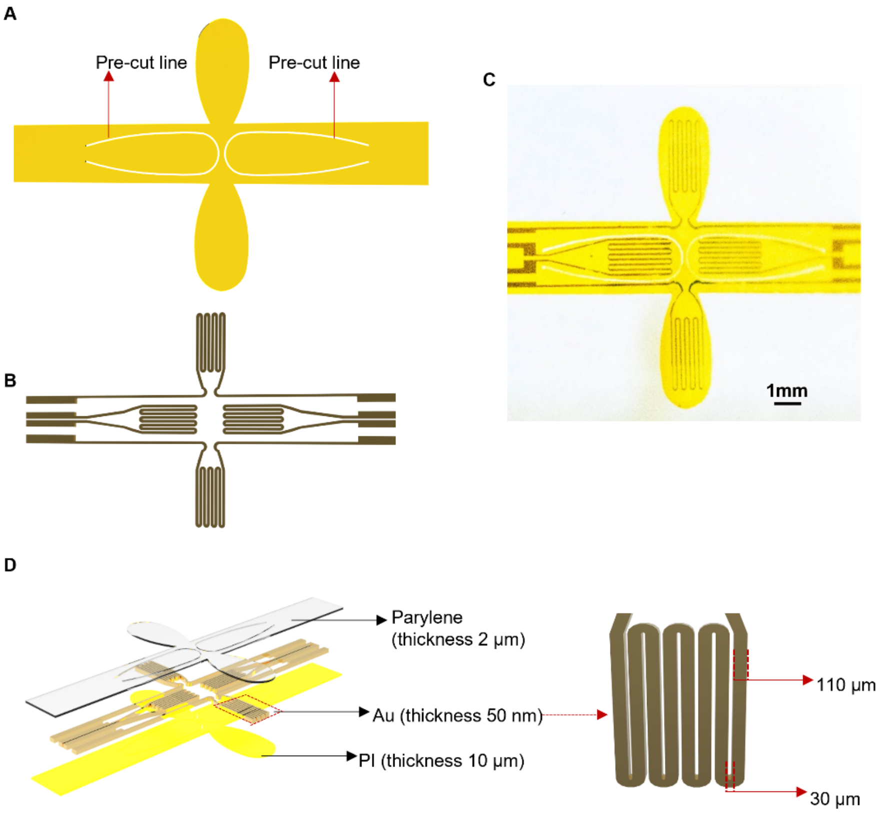

Fig. S18. The planar form of an epicardial bioelectronic system. (**A**) Pattern design of polyimide substrate. (**B**) Design of the four strain sensors made of Au serpentine resistors. (**C**) Optical image of a planar epicardial bioelectronic system with four strain sensors that are located on 4 respective petal-like cantilevers. (**D**) An exploded schematic view of the planar epicardial bioelectronic system.

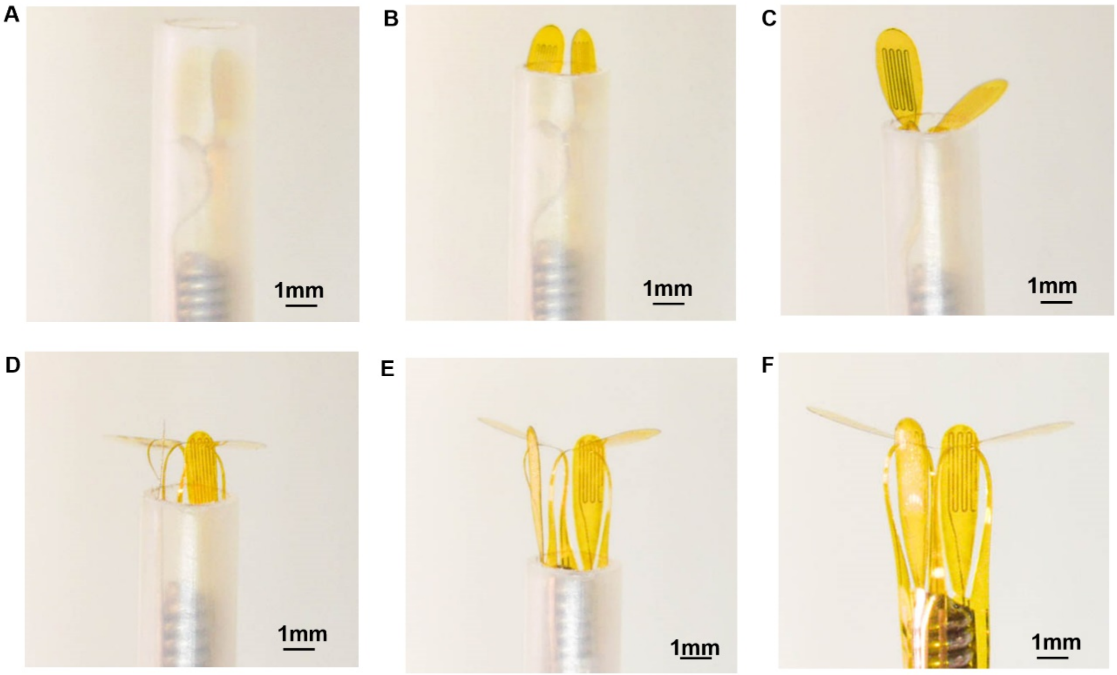

Fig. S19. The blooming process of the epicardial bioelectronic robot. (**A** to **F**) Optical images showing the blooming process of an epicardial bioelectronic robot. The final bloomed state has all four sensors spread out like a flower.

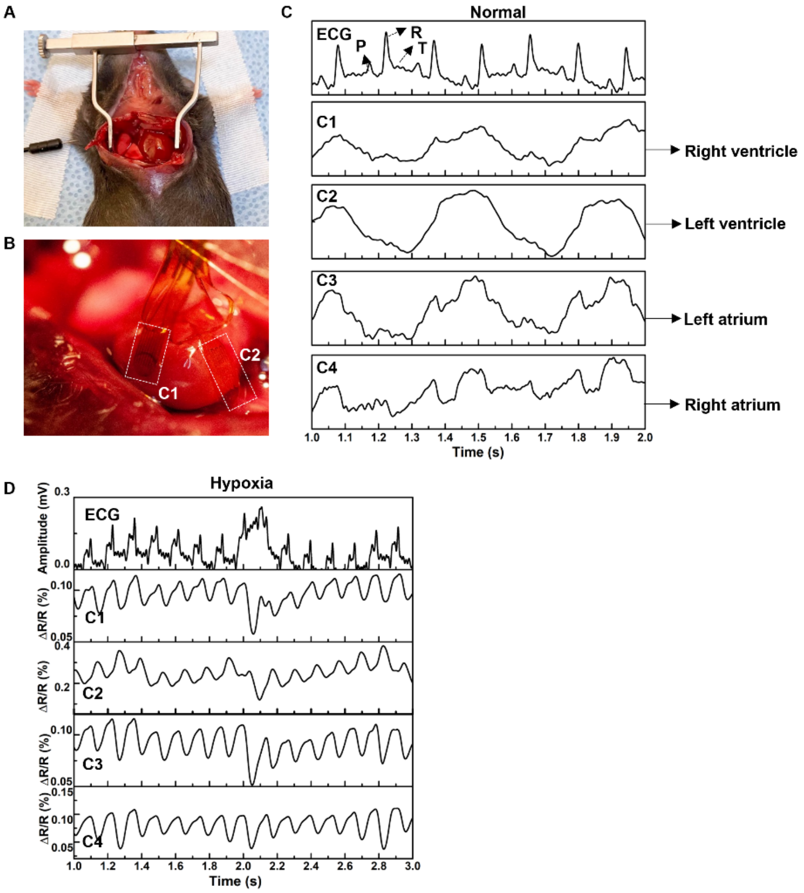

Fig. S20. The bloomable robot for cardiac deployment. (**A**) Optical image showing the animal study with the 3D epicardial bioelectronic robot engaged onto the cardiac surface. (**B**) The epicardial bioelectronic robot with a 3D flower-like architecture is attached to the end of the catheter and positioned on the mouse heart, which allows it to holistically monitor cardiac contractility using four different channels, corresponding to four separate sensors. Here, the sensor C1 and C2 are distributed on right ventricle (RV) and left ventricle (LV), respectively. (**C**) Representative measurements of an epicardial bioelectronic robot placed onto a living mouse heart under normal conditions (partial pressure of oxygen was 150 mmHg) and (**D**) hypoxia condition (partial pressure of oxygen was 50 mmHg).

Movie S1: 3D freestanding mesostructures via deterministic microfolding

REFERENCES AND NOTES

1. F. Zhang, S. Li, Z. Shen, X. Cheng, Z. Xue, H. Zhang, H. Song, K. Bai, D. Yan, H. Wang, Y. Zhang, Y. Huang, Rapidly deployable and morphable 3D mesostructures with applications in multimodal biomedical devices. *Proc. Natl. Acad. Sci. U.S.A.* **118**, e2026414118 (2021).

2. Y. Park, K. Kwon, S. S. Kwak, D. S. Yang, J. W. Kwak, H. Luan, T. S. Chung, K. S. Chun, J. U. Kim, H. Jang, H. Ryu, H. Jeong, S. M. Won, Y. J. Kang, M. Zhang, D. Pontes, B. R. Kampmeier, S. H. Seo, J. Zhao, I. Jung, Y. Huang, S. Xu, J. A. Rogers, Wireless, skin-interfaced sensors for compression therapy. *Sci. Adv.* **6**, eabe1655 (2020).

3. J. W. Phillips, A. Prominski, B. Tian, Recent advances in materials and applications for bioelectronic and biorobotic systems. *View* **3**, 20200157 (2022).

4. H. Kim, S.-K. Ahn, D. M. Mackie, J. Kwon, S. H. Kim, C. Choi, Y. H. Moon, H. B. Lee, S. H. Ko, Shape morphing smart 3D actuator materials for micro soft robot. *Mater. Today* **41**, 243–269 (2020).

5. K. Bai, X. Cheng, Z. Xue, H. Song, L. Sang, F. Zhang, F. Liu, X. Luo, W. Huang, Y. Huang, Y. Zhang, Geometrically reconfigurable 3D mesostructures and electromagnetic devices through a rational bottom-up design strategy. *Sci. Adv.* **6**, eabb7417 (2022).

6. H. Fu, K. Nan, W. Bai, W. Huang, K. Bai, L. Lu, C. Zhou, Y. Liu, F. Liu, J. Wang, M. Han, Z. Yan, H. Luan, Y. Zhang, Y. Zhang, J. Zhao, X. Cheng, M. Li, J. W. Lee, Y. Liu, D. Fang, X. Li, Y. Huang, Y. Zhang, J. A. Rogers, Morphable 3D mesostructures and microelectronic devices by multistable buckling mechanics. *Nat. Mater.* **17**, 268–276 (2018).

7. M. Humood, Y. Shi, M. Han, J. Lefebvre, Z. Yan, M. Pharr, Y. Zhang, Y. Huang, J. A. Rogers, A. A. Polycarpou, 3D mesostructures: Fabrication and deformation of 3D multilayered kirigami microstructures (Small 11/2018). *Small* **14**, 1870045 (2018).

8. X. Ning, X. Yu, H. Wang, R. Sun, R E Corman, H. Li, C. M. Lee, Y. Xue, A. Chempakasseril, Y. Yao, Z. Zhang, H. Luan, Z. Wang, W. Xia, X. Feng, R. H. Ewoldt, Y. Huang, Y. Zhang, J. A.

Rogers, Mechanically active materials in three-dimensional mesostructures. *Sci. Adv.* **4**, eaat8313 (2022).

9. C. El Helou, P. R. Buskohl, C. E. Tabor, R. L. Harne, Digital logic gates in soft, conductive mechanical metamaterials. *Nat. Commun.* **12**, 1633 (2021).

10. Y. Ling, W. Pang, J. Liu, M. Page, Y. Xu, G. Zhao, D. Stalla, J. Xie, Y. Zhang, Z. Yan, Bioinspired elastomer composites with programmed mechanical and electrical anisotropies. *Nat. Commun.* **13**, 524 (2022).

11. J. K. Park, K. Nan, H. Luan, N. Zheng, S. Zhao, H. Zhang, X. Cheng, H. Wang, K. Li, T. Xie, Y. Huang, Y. Zhang, S. Kim, J. A. Rogers, Remotely triggered assembly of 3D mesostructures through shape-memory effects. *Adv. Mater.* **31**, e1905715 (2019).

12. Y. Li, C. Luo, K. Yu, X. Wang, Remotely controlled, reversible, on-demand assembly and reconfiguration of 3D mesostructures via liquid crystal elastomer platforms. *ACS Appl. Mater. Interfaces* **13**, 8929–8939 (2021).

13. H. Luan, X. Cheng, A. Wang, S. Zhao, K. Bai, H. Wang, W. Pang, Z. Xie, K. Li, F. Zhang, Y. Xue, Y. Huang, Y. Zhang, Design and fabrication of heterogeneous, deformable substrates for the mechanically guided 3D assembly. *ACS Appl. Mater. Interfaces* **11**, 3482–3492 (2019).

14. Z. Yan, F. Zhang, F. Liu, M. Han, D. Ou, Y. Liu, Q. Lin, X. Guo, H. Fu, Z. Xie, M. Gao, Y. Huang, J. H. Kim, Y. Qiu, K. Nan, J. Kim, P. Gutruf, H. Luo, A. Zhao, K.-C. Hwang, Y. Huang, Y. Zhang, J. A. Rogers, Mechanical assembly of complex, 3D mesostructures from releasable multilayers of advanced materials. *Sci. Adv.* **2**, e1601014 (2016).

15. D. C. Kim, H. Yun, J. Kim, H. Seung, W. S. Yu, J. H. Koo, J. Yang, J. H. Kim, T. Hyeon, D. H. Kim, Three-dimensional foldable quantum dot light-emitting diodes. *Nat. Electron.* **4**, 671–680 (2021).

16. L. Skoric, D. Sanz-Hernández, F. Meng, C. Donnelly, S. Merino-Aceituno, A. Fernández-Pacheco, Layer-by-layer growth of complex-shaped three-dimensional nanostructures with focused electron beams. *Nano Lett.* **20**, 184–191 (2020).

17. Q. Geng, D. Wang, P. Chen, S.-C. Chen, Ultrafast multi-focus 3-D nano-fabrication based on two-photon polymerization. *Nat. Commun.* **10**, 2179 (2019).

18. B. X. E. Desbiolles, A. Bertsch, P. Renaud, Ion beam etching redeposition for 3D multimaterial nanostructure manufacturing. *Microsyst. Nanoeng.* **5**, 11 (2019).

19. J. K. Placone, A. J. Engler, Recent advances in extrusion-based 3D printing for biomedical applications. *Adv. Healthc. Mater.* **7**, 1701161 (2018).

20. G. Huang, Y. Mei, Assembly and self-assembly of nanomembrane materials—From 2D to 3D. *Small* **14**, 1703665 (2018).

21. A. M. Abdullah, X. Li, P. V. Braun, J. A. Rogers, K. J. Hsia, Kirigami-inspired self-assembly of 3D structures. *Adv. Funct. Mater.* **30**, 1909888 (2020).

22. H. Zhao, X. Cheng, C. Wu, T.-L. Liu, Q. Zhao, S. Li, X. Ni, S. Yao, M. Han, Y. Huang, Y. Zhang, J. A. Rogers, Mechanically guided hierarchical assembly of 3D mesostructures. *Adv. Mater.* **34**, 2109416 (2022).

23. H. Zhao, X. Cheng, C. Wu, T.-L. Liu, Q. Zhao, S. Li, X. Ni, S. Yao, M. Han, Y. Huang, Y. Zhang, J. A. Rogers, Controlled mechanical buckling for origami-inspired construction of 3D microstructures in advanced materials. *Adv. Funct. Mater.* **26**, 2629–2639 (2016).

24. X. Wang, X. Guo, J. Ye, N. Zheng, P. Kohli, D. Choi, Y. Zhang, Z. Xie, Q. Zhang, H. Luan, K. Nan, B. H. Kim, Y. Xu, X. Shan, W. Bai, R. Sun, Z. Wang, H. Jang, F. Zhang, Y. Ma, Z. Xu, X. Feng, T. Xie, Y. Huang, Y. Zhang, J. A. Rogers, Freestanding 3D mesostructures, functional devices, and shape-programmable systems based on mechanically induced assembly with shape memory polymers. *Adv. Mater.* **31**, e1805615 (2019).

25. Z. Yan, M. Han, Y. Shi, A. Badea, Y. Yang, A. Kulkarni, E. Hanson, M. E. Kandel, X. Wen, F. Zhang, Y. Luo, Q. Lin, H. Zhang, X. Guo, Y. Huang, K. Nan, S. Jia, A. W. Oraham, M. B. Mevis, J. Lim, X. Guo, M. Gao, W. Ryu, K. J. Yu, B. G. Nicolau, A. Petronico, S. S. Rubakhin, J. Lou, P. M. Ajayan, K. Thornton, G. Popescu, D. Fang, J. V. Sweedler, P. V. Braun, H. Zhang, R. G. Nuzzo, Y. Huang, Y. Zhang, J. A. Rogers, Three-dimensional mesostructures as high-

temperature growth templates, electronic cellular scaffolds, and self-propelled microrobots. **114**, E9455–E9464 (2017).

26. T. Castle, D. M. Sussman, M. Tanis, R. D. Kamien, Additive lattice kirigami. *Sci. Adv.* **2**, e1601258 (2022).

27. L. H. Dudte, E. Vouga, T. Tachi, L. Mahadevan, Programming curvature using origami tessellations. *Nat. Mater.* **15**, 583–588 (2016).

28. Y. Zhang, Z. Yan, K. Nan, D. Xiao, Y. Liu, H. Luan, H. Fu, X. Wang, Q. Yang, J. Wang, W. Ren, H. Si, F. Liu, L. Yang, H. Li, J. Wang, X. Guo, H. Luo, L. Wang, Y. Huang, J. A. Rogers, A mechanically driven form of Kirigami as a route to 3D mesostructures in micro/nanomembranes. *Proc. Natl. Acad. Sci. U.S.A.* **112**, 11757–11764 (2015).

29. H. Zhao, K. Li, M. Han, F. Zhu, A. Vázquez-Guardado, P. Guo, Z. Xie, Y. Park, L. Chen, X. Wang, H. Luan, Y. Yang, H. Wang, C. Liang, Y. Xue, R. D. Schaller, D. Chanda, Y. Huang, Y. Zhang, J. A. Rogers, Buckling and twisting of advanced materials into morphable 3D mesostructures. *Proc. Natl. Acad. Sci. U.S.A.* **116**, 13239–13248 (2019).

30. Z. Yan, M. Han, Y. Yang, K. Nan, H. Luan, Y. Luo, Y. Zhang, Y. Huang, J. A. Rogers, Deterministic assembly of 3D mesostructures in advanced materials via compressive buckling: A short review of recent progress. *Extrem. Mech. Lett.* **11**, 96–104 (2017).

31. J. E. Huddy, M. S. Rahman, A. B. Hamlin, Y. Ye, W. J. Scheideler, Transforming 3D-printed mesostructures into multimodal sensors with nanoscale conductive metal oxides. *Cell Rep. Phys. Sci.* **3**, 100786 (2022).

32. Y. Zhang, F. Zhang, Z. Yan, Q. Ma, X. Li, Y. Huang, J. A. Rogers, Printing, folding and assembly methods for forming 3D mesostructures in advanced materials. *Nat. Rev. Mater.* **2**, 17019 (2017).

33. Y. Q. Fu, J. K. Luo, A. J. Flewitt, S. E. Ong, S. Zhang, H. J. Du, W. I. Milne, Microactuators of free-standing TiNiCu films. *Smart Mater. Struct.* **16**, 2651–2657 (2007).

34. Y. Li, H. Yu, K. Yu, X. Guo, X. Wang, Reconfigurable three-dimensional mesotructures of spatially programmed liquid crystal elastomers and their ferromagnetic composites. *Adv. Funct. Mater.* **31**, 2100338 (2021).

35. H. Fu, K. Bai, Y. Huang, Y. Zhang, Recent progress of morphable 3D mesostructures in advanced materials. *J. Semicond.* **41**, 041604 (2020).

36. S. C. K. Ko, R. D. Murch, Compact integrated diversity antenna for wireless communications. *IEEE Trans. Antennas Propag.* **49**, 954–960 (2001).

37. J.-Y. Jan, L.-C. Tseng, Small planar monopole antenna with a shorted parasitic inverted-L wire for wireless communications in the 2.4-, 5.2-, and 5.8-GHz bands. *IEEE Trans. Antennas Propag.* **52**, 1903–1905 (2004).

38. D. Mitra, D. Das, S. R. B. Chaudhuri, Miniaturization of meander line slot antenna, in *2015 IEEE-APS Topical Conference on Antennas and Propagation in Wireless Communications (APWC)* (2015), pp. 1255–1257.

39. H. J. Visser, Analytical equations for the analysis of folded dipole array antennas, in *2008 38th European Microwave Conference* (2008), pp. 706–709.

40. J. W. Kwak, M. Han, Z. Xie, H. U. Chung, J. Y. Lee, R. Avila, J. Yohay, X. Chen, C. Liang, M. Patel, I. Jung, J. Kim, M. Namkoong, K. Kwon, X. Guo, C. Ogle, D. Grande, D. Ryu, D. H. Kim, S. Madhvapathy, C. Liu, D. S. Yang, Y. Park, R. Caldwell, A. Banks, S. Xu, Y. Huang, S. Fatone, J. A. Rogers, Wireless sensors for continuous, multimodal measurements at the skin interface with lower limb prostheses. *Sci. Transl. Med.* **12**, 1–14 (2020).

41. M. Fallahpour, R. Zoughi, Antenna miniaturization techniques: A review of topology- and material-based methods. *IEEE Antennas Propag. Mag.* **60**, 38–50 (2018).

42. P. Anacleto, H. Dinis, J. Fernandes, P. M. Mendes, Design and characterization of 3-D self-folded microantennas for implantable microdevices. *IEEE Trans. Antennas Propag.* **68**, 2031–2039 (2020).

43. A. Puschmann, Z. K. Wszolek, Diagnosis and treatment of common forms of tremor. *Semin. Neurol.* **31**, 65–77 (2011).

44. X. Zheng, A. V. Campos, J. Ordieres-Meré, J. Balseiro, S. L. Marcos, Y. Aladro, Continuous monitoring of essential tremor using a portable system based on smartwatch. *Front. Neurol.* **8**, 96 (2017).

45. K. R. Chaudhuri, M. Buxton-Thomas, V. Dhawan, R. Peng, C. Meilak, D. J. Brooks, Long duration asymmetrical postural tremor is likely to predict development of Parkinson's disease and not essential tremor: Clinical follow up study of 13 cases. *J. Neurol. Neurosurg. Psychiatry* **76**, 115–117 (2005).

46. D. M. Halliday, B. A. Conway, S. F. Farmer, U. Shahani, A. J. Russell, J. R. Rosenberg, Coherence between low-frequency activation of the motor cortex and tremor in patients with essential tremor. *Lancet* **355**, 1149–1153 (2000).

47. Y. Moshkovitz, E. Kaluski, O. Milo, Z. Vered, G. Cotter, Recent developments in cardiac output determination by bioimpedance: Comparison with invasive cardiac output and potential cardiovascular applications. *Curr. Opin. Cardiol.* **19**, 229–237 (2004).

48. T. M. Hoffman, G. Wernovsky, A. M. Atz, T. J. Kulik, D. P. Nelson, A. C. Chang, J. M. Bailey, A. Akbary, J. F. Kocsis, R. Kaczmarek, T. L. Spray, D. L. Wessel, Efficacy and safety of milrinone in preventing low cardiac output syndrome in infants and children after corrective surgery for congenital heart disease. *Circulation* **107**, 996–1002 (2003).

49. S. D. Anker, M. Borggrefe, H. Neuser, M.-A. Ohlow, S. Röger, A. Goette, B. A. Remppis, K.-H. Kuck, K. B. Najarian, D. D. Gutterman, B. Rousso, D. Burkhoff, G. Hasenfuss, Cardiac contractility modulation improves long-term survival and hospitalizations in heart failure with reduced ejection fraction. *Eur. J. Heart Fail.* **21** 1103–1113 (2019).

50. F. A. D. Gutterman, Cardiac contractility modulation: A novel approach for the treatment of heart failure. *Heart Fail. Rev.* **21**, 645–660 (2016).

51. M. Santos, D. W. Kitzman, K. Matsushita, L. Loehr, C. A. Sueta, A. M. Shah, Prognostic importance of dyspnea for cardiovascular outcomes and mortality in persons without prevalent cardiopulmonary disease: The atherosclerosis risk in communities study. *PLOS ONE* **11**, e0165111 (2016).

52. M. Santos, D. W. Kitzman, K. Matsushita, L. Loehr, C. A. Sueta, A. M. Shah, Rat infarct model of myocardial infarction and heart failure. *J. Card. Fail.* **1**, 169–177 (1995).

53. O. Tarnavski, J. R. McMullen, M. Schinke, Q. Nie, S. Kong, S. Izumo, Mouse cardiac surgery: Comprehensive techniques for the generation of mouse models of human diseases and their application for genomic studies. *Physiol. Genomics* **16**, 349–360 (2004).

54. M. Kumar, E. R. Kasala, L. N. Bodduluru, V. Dahiya, D. Sharma, V. Kumar, M. Lahkar, Animal models of myocardial infarction: Mainstay in clinical translation. *Regul. Toxicol. Pharmacol.* **76**, 221–230 (2016).

55. W. Oldendorf, W. Oldendorf Jr. Advantages and disadvantages of MRI, in *Basics of Magnetic Resonance Imaging*, W. Oldendorf, W. Oldendorf, Eds. (Springer, 1988). pp. 125–138.

56. R. A. Nishimura, F. A. Miller Jr., M. J. Callahan, R. C. Benassi, J. B. Seward, A. J. Tajik, Doppler echocardiography: Theory, instrumentation, technique, and application. *Mayo Clin. Proc.* **60**, 321–343 (1985).

57. Y. Park, C. K. Franz, H. Ryu, H. Luan, K. Y. Cotton, J. U. Kim, T. S. Chung, S. Zhao, A. Vazquez-Guardado, D. S. Yang, K. Li, R. Avila, J. K. Phillips, M. J. Quezada, H. Jang, S. S. Kwak, S. M. Won, K. Kwon, H. Jeong, A. J. Bandodkar, M. Han, H. Zhao, G. R. Osher, H. Wang, K. H. Lee, Y. Zhang, Y. Huang, J. D. Finan, J. A. Rogers, Three-dimensional, multifunctional neural interfaces for cortical spheroids and engineered assembloids. *Sci. Adv.* **7**, eabf9153 (2022).

58. S. M. Won, H. Wang, B. H. Kim, K. H. Lee, H. Jang, K. Kwon, M. Han, K. E. Crawford, H. Li, Y. Lee, X. Yuan, S. B. Kim, Y. S. Oh, W. J. Jang, J. Y. Lee, S. Han, J. Kim, X. Wang, Z. Xie, Y.

Zhang, Y. Huang, J. A. Rogers, Multimodal sensing with a three-dimensional piezoresistive structure. *ACS Nano* **13**, 10972–10979 (2019).

59. H. Luan, Q. Zhang, T.-L. Liu, X. Wang, S. Zhao, H. Wang, S. Yao, Y. Xue, J. W. Kwak, W. Bai, Y. Xu, M. Han, K. Li, Z. Li, X. Ni, J. Ye, D. Choi, Q. Yang, J.-H. Kim, S. Li, S. Chen, C. Wu, D. Lu, J.-K. Chang, Z. Xie, Y. Huang, J. A. Rogers, Complex 3D microfluidic architectures formed by mechanically guided compressive buckling. *Sci. Adv.* **7**, eabj3686 (2022).

60. J. M. Lilly, S. C. Olhede, Higher-order properties of analytic wavelets. *IEEE Trans. Signal Process.* **57**, 146–160 (2009).

61. Z. Xie, R. Avila, Y. Huang, J. A. Rogers, Flexible and stretchable antennas for biointegrated electronics. *Adv. Mater.* **32**, 1902767 (2020).

62. T. S. Bird, Definition and misuse of return loss [Report of the Transactions Editor-in-Chief]. *IEEE Antennas Propag. Mag.* **51**, 166–167 (2009).
